# Experimental manipulation of population density in a wild bird alters social structure but not patch discovery rate

**DOI:** 10.1016/j.anbehav.2023.12.010

**Published:** 2024-03

**Authors:** Kristina B. Beck, Charlotte E. Regan, Keith McMahon, Sam Crofts, Ella F. Cole, Josh A. Firth, Ben C. Sheldon

**Affiliations:** a Edward Grey Institute, Department of Biology, University of Oxford, Oxford, U.K.; b School of Biology, University of Leeds, Leeds, U.K.

**Keywords:** experiment, great tit, information transmission, population density, social network

## Abstract

Population density is a fundamental ecological feature influencing the opportunity for social encounters between individuals. Hence, density can impact various population processes such as social transmission. While the density dependence of disease spread has been studied extensively, we know little about how variation in density influences information transmission. If high densities lead to more social connections, information may spread more rapidly. Here, we experimentally manipulated local population density in great tits, *Parus major*, and investigated the effects on individuals’ acquisition of information on novel food patches. We manipulated density by assigning individuals to either a high- or low-density treatment using automated bird feeders in the wild. We first show how our approach successfully led to changes in local population density. Next, we examine how the manipulation changed the local and individual social environment. At the local level, high-density locations resulted on average in denser and more clustered social networks compared to low-density locations. At the individual level, birds assigned to the high-density treatment had on average more social connections and more central network positions than birds in the low-density treatment. However, despite the effect on network structure, we found no evidence that the density manipulation influenced an individuals’ likelihood, or speed, of locating novel food patches. Birds in the low-density treatment still spent a large proportion of time foraging at the high-density location. Thus, the manipulation did not lead to a strict segregation between birds of the different treatments, which may be one explanation for the absence of an effect on patch discovery.

Population density, defined as the number of individuals in a given geographical unit, can fundamentally shape the opportunity for, and frequency of, social encounters between individuals. Commonly, the rate of social encounters between individuals increases with increasing population density (Albery, 2022; Hutchinson & Waser, 2007; Sanchez & Hudgens, 2015; Vander Wal et al., 2014). As such, population density influences various ecological processes ranging from competition to mating and the social transmission of diseases (Hopkins et al., 2020; Jirotkul, 1999; Kokko & Rankin, 2006). For example, a large body of research has examined density dependence of the transmissibility of diseases and parasites whereby higher densities often facilitate transmission (Anderson, 1982; Brunner et al., 2017; Hopkins et al., 2020; Colman et al., 2021; for a current review see Albery, 2022). Population density and its consequences for social encounter rates between individuals may also impact other forms of social transmission such as the spread of novel information across a population. However, while the density dependence of disease transmission has been studied extensively, our knowledge of how density impacts the transmission of information remains superficial.

Animals use social information in various contexts (Hoppitt & Laland, 2013; Whiten, 2021). The transfer of social information, where naïve individuals copy the behaviour of knowledgeable others, often requires close proximity encounters between individuals. Therefore, the social connections between individuals can predict when and where information flows (Hasenjager et al., 2021). Past research has demonstrated how social network structure profoundly impacts the transmission of social information. For instance, global properties of social networks such as the density of social connections can influence the likelihood and efficiency of information flow whereby networks with a higher density of social connections facilitate transmission (Pasquaretta et al., 2014). At an individual level, variation in the number and strength of social connections to conspecifics determines whether and when individuals have access to information. As such, individuals with more central network positions and more social connections are often more likely to acquire information and to do so faster (Aplin et al., 2012; Kulahci et al., 2016; Schakner et al., 2017).

Higher population densities can lead to more opportunities for social encounters (Hein & McKinley, 2013; Sanchez & Hudgens, 2015; Vander Wal et al., 2014) and larger group sizes (Pépin & Gerard, 2008; Vander Wal et al., 2013). Consequently, population density can fundamentally affect the social environment. For instance, if increased densities lead to more social encounters (Hu et al., 2013), individuals may have on average more social connections compared to individuals at lower densities. On the population level, increased encounter rates may lead to more realized social connections over the total number of possible social connections (i.e. increased network densities). However, animals are often territorial or preferably associate with specific individuals while avoiding others in which case increases in population density may not necessarily change the probability of social encounters (Albery, 2022; Hutchinson & Waser, 2007). Importantly, if population density affects the social connections between individuals, it can affect ecological processes reliant on social connections such as the transmission of social information. For example, if higher densities increase the social connections between individuals, novel information may spread faster compared to populations with low densities because of the increased opportunities for information exchange. Yet, the role of population density in shaping individual connectivity and population social structure, and subsequently social information transmission, is largely unexplored.

Investigating the relationship between population density, social structure and information transmission in the wild is challenging. This is because it requires tracking the social encounters of multiple individuals at the same time and following the transmission of novel information. Further, to draw causal inferences, experimental studies manipulating population densities and seeding novel information are required. Past research demonstrated that experimental studies can be used to examine the spread of novel information by, for instance, establishing novel resource patches (Aplin et al., 2012; Atton et al., 2014; Snijders et al., 2021) and few studies experimentally manipulated group size to examine the effects on various processes including information transmission (Pitcher et al., 1982; Snijders et al., 2021). However, research experimentally manipulating ecological factors such as population density in the wild are scarce.

We addressed the challenges of examining the causal relationships between ecological factors and information transmission by experimentally manipulating the density of local subpopulations in a wild songbird the great tit, *Parus major*. By controlling the number of individuals that could access automated feeding stations, we determined (1) whether the experimentally imposed treatments led to the predicted changes in local population density (i.e. low versus high density) on a local and individual scale, (2) what consequences population density had for the local social network structure and individual sociality, and (3) how population density impacted the information transmission of novel food patches. More specifically, we first manipulated local population density by assigning individual great tits to either a low- or high-density treatment, aiming to create a low- and high-density location. Second, we examined how our manipulations affected characteristics of the global (i.e. network density, global clustering coefficient and average edge weight) and individual (i.e. average edge weight, weighted degree, weighted clustering coefficient and weighted eigenvector centrality) social environment using social network analysis. Third, we asked how the treatment (low versus high density) influenced the probability and speed of acquiring information about a novel food source.

Great tits forage in fission–fusion flocks during winter (Ekman, 1989). We predicted that at the high-density treatment, increased opportunities for social encounters at the feeder should lead to denser and more clustered social networks but lead to lower average edge weights. This is because we expect that individuals have strong connections to a few preferred conspecifics but that the increase in density will lead to more random social encounters whose connections should be less strong leading to on average lower edge weights. Consequently, individuals assigned to the high-density treatment should have a higher social connectivity (i.e. higher weighted degree, clustering and eigenvector centrality) but on average less strong social connections (i.e. lower average edge weights) compared to individuals assigned to the low-density treatment. Great tits frequently use social information to locate novel foraging patches (Aplin et al., 2012; Firth et al., 2016) and individuals with a higher social connectivity are more likely to discover novel food sources (Aplin et al., 2012). Thus, if population density influences social connectivity, we expected that individuals in the high-density treatment should be more likely to locate the provided novel food patches and to be faster to do so.

## Methods

### Study System

This study was conducted in Wytham woods (Savill et al., 2011), Oxfordshire, U.K. (51°46′N, 1°20′W) on a population of great tits. Great tits are small passerine birds that form socially monogamous pair bonds during the breeding season and forage with conspecifics, as well as other species, in fission–fusion flocks during winter (Ekman, 1989). Birds were either caught during winter using mist-nets or during spring in nestboxes and fitted with a unique metal leg ring from the British Trust for Ornithology (BTO), and a plastic leg ring with an inbuilt unique passive integrated transponder (PIT) tag. Great tits use social information to locate novel food patches (Aplin et al., 2012, 2015; Farine et al., 2015), and experimental manipulations of social structure using RFID systems have successfully been performed in the past (Firth et al., 2016; Firth & Sheldon, 2015).

### Experimental Manipulation of Local Population Density

**Figure 1:**
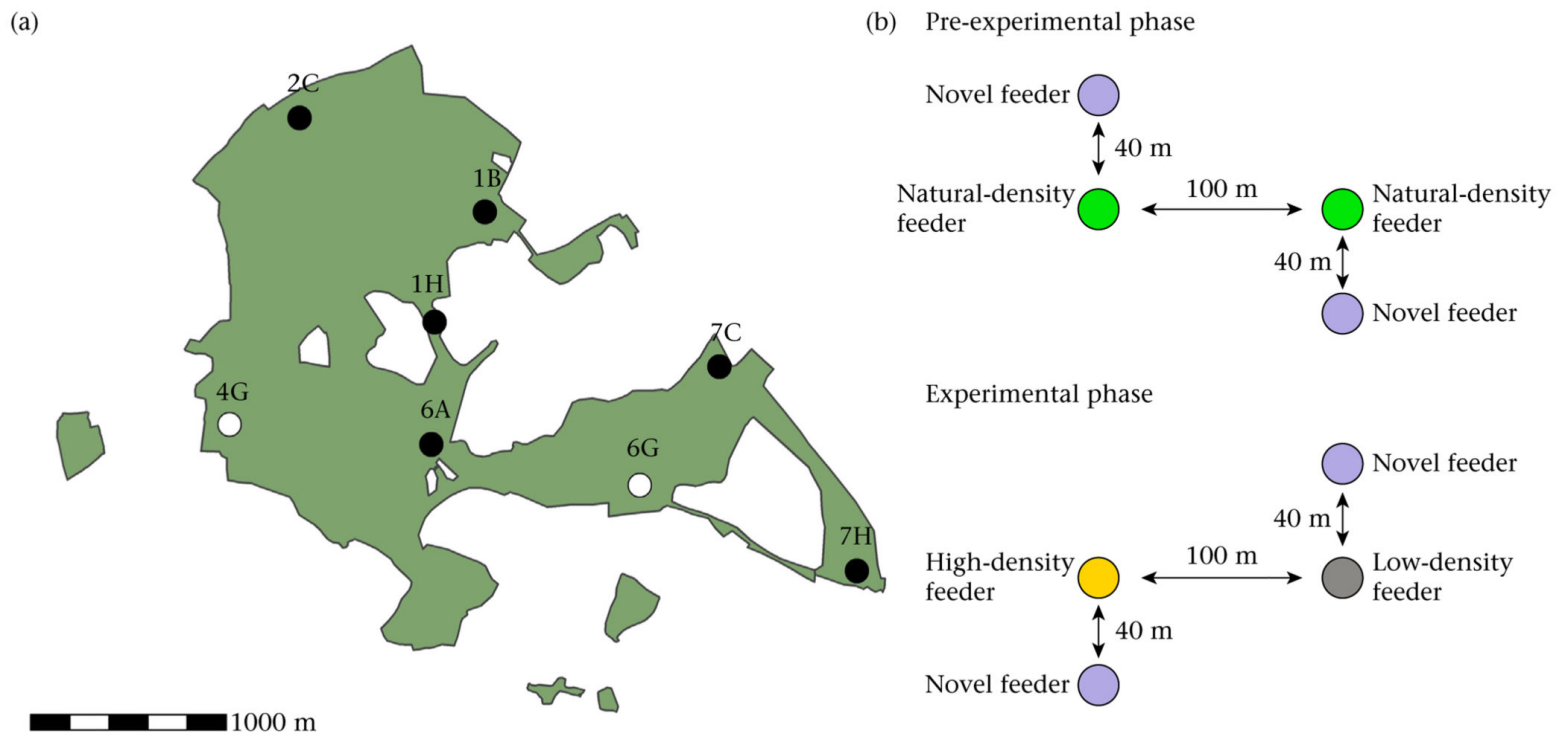
(a) Locations of experimental sites within the study site. Six sites shown in black (1B, 1H, 2C, 6A, 7C, 7H) functioned as experimental sites and two sites shown in white as control sites (4G, 6G). (b) The set-up within each site during the density manipulation and the patch discovery experiment. Green dots show the two feeders prior to the density manipulation. The orange dot represents the high-density feeder during the experiment, the grey dot, the low-density feeder. Blue dots represent the novel feeders, which were positioned at any location within 40 m of the established feeders (i.e. the natural/low-/high-density feeder) phasing in opposite directions (i.e. if one novel feeder was placed 40 m north of the low-density feeder, the second novel feeder was placed 40 m south of the high-density feeder).

The experiment took place from January to March in 2021. We used ‘selectivé feeders to record visits from birds and to subsequently manipulate local population density. Each of the feeders had one access hole with a flap and an inbuilt RFID antenna within the perch. All birds equipped with a PIT tag that landed on the perch were recorded, and the time and date of visits stored on an SD card. The flap was programmed to open only for specified PIT-tagged individuals which allowed us to manipulate local densities. We set up eight replicate sites across our study site (Fig. 1a), each with two feeders approximately 100 m apart (Fig. 1b) following set-ups of previous work using selective feeders (Firth et al., 2016; Firth & Sheldon, 2015). Sites were at least 500 m apart to minimize movement of birds between them. All feeders provided sunflower seeds. Within six experimental sites, one feeder was assigned as the low-density treatment and the other as the high-density treatment (Fig. 1b) and two sites were randomly chosen as control sites. Prior to starting the experimental manipulation of local population density, we collected data on the birds’ visits for approximately 2 weeks. In this pre-experimental phase, both selective feeders at each site allowed all birds with a PIT tag access 24 h per day. At each site, we then quantified the number of visits to each of the two selective feeders. Then, at half of the experimental sites, the feeder with the majority of visits prior to the experiment was assigned to the high-density treatment (and the other feeder to the low-density treatment). At the other half, the feeder with the majority of visits was assigned to the low-density treatment (and the other feeder to the high-density treatment). Finally, of all the great tits recorded at least 100 times during the pre-experimental period (75%; Fig. A1) at a given site, we randomly assigned 20% to the low-density treatment and excluded these 20% from accessing the high-density treatment. We selected the low-density birds only from those recorded at least 100 times to avoid selecting birds that had only been transient at the site (following Regan et al., 2022; see also Fig. A1 and corresponding summary statistics in the figure legend). Thus, at the low-density feeder, only the assigned 20% could access food, whereas at the high-density feeder all PIT-tagged birds could access food, except the 20% chosen for the low-density treatment. Visits to a feeder by PIT-tagged birds were always recorded, i.e. if a bird assigned to the low-density treatment visited the high-density feeder it would be unable to access food, but its visit would be recorded. At the two control sites, every PIT-tagged bird could access food anytime. The experimental manipulation was in place for 6 weeks. Feeders were visited at least three times a week to ensure that food was available ad libitum across the experiment, to check feeder performance and to collect data.

### Patch Discovery Experiment

We aimed to investigate how changes in the local population density influenced the likelihood and speed of discovering novel resources. Therefore, we set up a novel food ‘patch’ near to each selective feeder and examined the likelihood and timing of discovery. As a novel food source, we placed a new feeder near to each of the two already present feeders (approximately 40 m, which is far enough to be out of sight for birds foraging at the already present feeders and close enough to allow the majority of birds to discover the food source within a day) at the experimental and control sites. Within each site, novel feeders were placed in opposite directions, that is, if one novel feeder was placed 40 m north of the low-density feeder, the second novel feeder was placed 40 m south of the high-density feeder (Fig. 1b). We performed a patch discovery experiment both prior to and during the density manipulation. For each patch discovery trial, we selected random, novel locations with similar vegetation structure, but with the same general arrangement (that is 40 m from the selective feeders, facing in opposite directions, see Fig. 1b). The novel feeders provided the same food as the selective feeders but were programmed to allow access to all PIT-tagged birds. Novel feeders were the same design as the selective feeders to avoid any effects of neophobia and were set up either the day before the experiment after sunset or on the day of the experiment before sunrise. Novel feeders were then in place for 1 day to record the arrivals of individuals.

### Ethical Note

The experiment was conducted in accordance with the ASAB/ABS guidelines (Buchanan et al., 2012) and was subject to review by the local ethical review committee of the Department of Biology, University of Oxford (Reference number: APA/1/5/ZOO/NASPA/Sheldon/BehaviouralContagion). Birds were caught, ringed and equipped with PIT-tags by experienced ringers under BTO licences.

### Social Networks Construction

Following previous work, we created social networks based on the foraging associations of PIT-tagged great tits. Records of visits will typically consist of periods with high activity and periods of low activity because great tits forage in flocks. We used the package ‘asnipé (Farine, 2013) in R (R Core Team, 2020; https://www.r-project.org/) to first detect events of feeding activity, second to cluster these in nonoverlapping flocking events, and finally to assign individuals to the events they most likely belong to using Gaussian mixture models (Psorakis et al., 2012). We defined an ‘association’ as two birds co-occurring in the same flock. We created undirected social networks with connections (edges) weighted using the simple ratio index (SRI; Cairns & Schwager, 1987). The SRI describes the proportion of observations of two individuals in which they were seen together, and ranges from 0 (never observed foraging in the same flock) to 1 (always observed foraging together in the same flock). Social networks inferred in this way from the data have been shown to be important and meaningful for various ecological processes within this population such as information transmission, reproduction and spatial breeding settlement (Aplin et al., 2015; Culina et al., 2015; Farine et al., 2015; Firth et al., 2015; Firth & Sheldon, 2016).

For the site level analyses, we created daily social networks for each feeder (i.e. low- versus high-density feeder) separately. We then calculated three global network metrics using the R package ‘igraph’ (Csardi & Nepusz, 2006): network density (unweighted) which describes how well individuals in the network are connected; the average edge weight which describes how strong the existing connections are (i.e. how much time individuals spent together foraging); and the global clustering coefficient (unweighted) which describes the extent to which nodes (i.e. individuals) tend to cluster together (Csardi & Nepusz, 2006). They are calculated, respectively, as the number of existing connections divided by all potential connections; the average of all nonzero edge weights; and the ratio of triangles and connected triples in the network.

For the individual level analyses, we created one social network for each site (i.e. pair of selective feeders) and period (prior to or during the experiment). We then calculated the average flock size, and four social network metrics for each individual. The four metrics assessed included the weighted degree which measures the number and strength of an individual’s connections, average association strength which measures the average strength of an individual’s social connections, weighted clustering coefficient which measures the extent to which an individual is ‘tightly’ clustered within its group and the weighted eigenvector centrality which extends the measure of weighted degree by also measuring the connectedness of an individual’s associates.

### Social assortment

We examined whether individuals assorted based on their assigned treatment, that is whether ‘high-density birds’ spend more time foraging together with ‘high-density birds’ than expected, and whether ‘low-density birds’ spend more time foraging together with ‘low-density birds’ than expected. To test this, we created social networks for each site and period, and calculated the assortativity coefficient (Newman, 2003) from the weighted associations using the R package ‘assortnet’ (Farine, 2014). The resulting assortment scores range from 1 (perfect assortment: all edges are between like individuals) to −1 (disassortment; all edges between unlike individuals), where a value of 0 would indicate random associations of ‘low’ and ‘high’ density birds. In addition, we created daily social networks and again calculated the assortativity coefficient. Here, we excluded days where fewer than two individuals of each treatment were present.

### Statistical Analysis

#### Site level changes

We predicted that the experimental changes in local population density would lead to a higher activity and number of individuals visiting at the high-density location compared to the low-density location. Therefore, we examined whether our experimental manipulation led to the predicted changes at the low- and high-density feeder within each site by exploring three different density measures: the daily proportion of recordings at each feeder, the total number of individuals recorded on a given day and the daily average flock size. Owing to technical issues, feeders occasionally failed to record data. Therefore, we excluded days on which one of the two feeders at each site was not recording data for our subsequent analysis (across all sites this was the case for about 22% of days). We fitted generalized linear mixed models (GLMM) with a binomial error structure (logit-link function) for the analysis on the proportion of daily visits to each feeder which was inferred from the number of recordings to the low- and high-density feeder compared to the total number of recordings at the corresponding experimental site. We fitted GLMMs with a Poisson error structure for the total number of individuals, and linear mixed models (LMM) for the average flock size. For all models, we included the period (pre versus during experimental phase) in interaction with the treatment (low- versus high-density feeder) and experimental day (defined as subsequent day within each period to account for any temporal trends within each experimental period) as explanatory variables. We included the site identity as a random effect and incorporated a first-order autoregressive pattern (AR1) for feeder identity nested within each site to account for temporal autocorrelation. All models were fitted using the ‘glmmTMB’ R package (Magnusson et al., 2017). Finally, we conducted post hoc comparisons between combinations of period and treatment using estimated marginal means using the ‘emmeans’ R package (Lenth et al., 2019). Pairwise comparisons between all factor levels were performed using Tukey-adjusted comparisons and *P* values were adjusted for multiple comparisons. This allowed us to examine whether density increased/decreased for the high-/low-density feeder in response to the experimental manipulation (within-feeder comparisons), and whether there was any difference in density between the low- and high-density feeder prior to and during the experimental manipulation (between-feeder comparisons).

Finally, we examined three daily network metrics characterizing the global network structure at each feeder: network density, the average edge weight and the global clustering coefficient. For the analysis, we removed networks where fewer than three individuals were connected (final data distribution of network sizes: minimum daily network size = 3, maximum = 43, mean = 17.8, see Fig. A2). We fitted LMMs for the response variables ‘network density’, ‘average edge weight’ (which was transformed by taking the inverse) and clustering coefficient (square transformed). The same explanatory variables were fitted as described above. As random intercept, we only included site identity because we detected no significant signs of temporal autocorrelation. We perfomed pairwise comparisons as described above.

#### Individual level changes

We aimed to examine whether the density experienced by individuals increased/decreased for the high-/low-density individuals in response to the experimental manipulation (within-individual comparisons), and whether there was any difference in density between the low- and high-density individuals prior to and during the experimental manipulation (between-individual comparisons). For our analyses on the individual level, we only selected birds that were recorded at least 100 times (following Regan et al., 2022) in each experimental period at a respective site (*N*_total_ = 162, *N*_control_ = 32, *N*_low_ = 36, *N*_high_ = 97 individuals, one bird experienced both low and high treatment, two birds visited an experimental and control site). First, we examined whether individuals significantly increased their visits to the feeder to which they had been assigned. For each individual, we calculated the number of visits to the low- and high-density feeder within each period. We then fitted a GLMM with the number of visits to the assigned feeder and the number of visits to the unassigned feeder as response variable (modelled with a binomial error structure). As explanatory variables, we included period (pre versus during experimental phase) in interaction with the treatment to which individuals had been assigned (low versus high). We included individual identity and site identity as random effects. In addition, we examined whether the ‘perceived’ density of individuals while foraging changed according to their assigned treatment. To do so, we fitted an LMM including mean flock size calculated for each individual and period as dependent variable. We included the same fixed and random effects as described above and conducted pairwise comparisons between all factor levels of period and treatment.

Finally, we investigated whether our experimental manipulation led to changes in individuals’ social network positions. We fitted LMMs including ‘weighted degreé, ‘average association strength’ and square transformed ‘weighted eigenvector centrality’ as response variables, and a GLMM for weighted clustering coefficient using a beta error structure. We included the same fixed and random effects as described above and conducted pairwise comparisons between all factor levels of period and treatment. We used network permutations to account for the nonindependence of social network data (i.e. individuals’ social network positions are dependent on one another (Whitehead, 2008)). Specifically, we performed a node permutation where the identity of each individual (i.e. node) in the network was randomized (Whitehead, 2008). Therefore, the overall social network structure for each experimental period remained constant but the social network positions that each individual occupied was altered. After each permutation, we re-ran the models described above and stored the estimated coefficients of each pairwise comparison. We repeated this process 1000 times. We inferred statistical significance of each pairwise comparison by comparing the generated null distribution of the coefficients for each pairwise comparison from the 1000 permutations to the coefficients for each pairwise comparison of the observed data. A *P* value <0.05 indicates that the observed coefficient lays outside of the 95% range of the null distribution for a given pairwise comparison (i.e. below the bottom 2.5% or above the top 97.5%).

Although our experimental sites were set in positions at least 500 m apart, a small minority of birds were recorded at more than one site and experienced different treatments (2%, see sample sizes above). For example, a bird assigned to the low-density treatment at site x may have moved after a few days to site y, experiencing the high-density treatment. However, because all our analyses were performed on the site level, that is each individual’s foraging behaviour and social associations at a respective site were considered and not the overall experienced social associations across all sites, we decided to include those individuals in our analysis. Further, by only selecting individuals with at least 100 recordings at each site and for each experimental period, we ensured that we only included birds that had experienced a given treatment for an extended period of time.

### Patch discovery experiment

We investigated whether an individual’s probability of discovering any novel feeder (regardless of whether it was the one placed next to the low- or high-density feeder) was predicted by its assigned treatment (access at the low- or high-density feeder). For our analyses, we only included birds recorded at the selective feeders on the day of the patch discovery experiment and that had been recorded at least 100 times across the density manipulation period (*N* = 148). We fitted a GLMM with a binomial error structure, including whether a bird discovered a novel feeder (yes/no) within the corresponding experimental period (pre/during the experiment) as the response variable. As explanatory variable, we fitted the treatment (individual assigned to high- or low-density treatment) in interaction with the period (pre/during the experiment). We included individual identity and site identity as random effects. Finally, for all individuals that did discover a novel feeder, we examined whether the order and latency in which they discovered the novel feeder were related to the density treatment. For the order of discovery, we defined birds visiting within 10 min of each other as discovering at the same time following previous work (Aplin et al., 2012; Firth et al., 2016). The latency of discovery was estimated as the time (s) from the start of the patch discovery experiment until the birds’ first arrival. We fitted LMMs with the log-transformed order of arrival and square root transformed latency as the response variable and included period and experimental treatment as fixed effects. Further, we included individual identity and site identity as random effects.

All data manipulation and analyses were carried out using R version 4.3.0. (R Core Team, 2020) and model fit was checked using graphical methods (e.g. qq plot of residuals, fitted values versus residuals).

## Results

We recorded in total 334 great tits across the study period (*N*_control_ = 95, *N*_low_ = 43 individuals, *N*_high_ = 239) of which six experienced both experimental treatments (because low-density birds moved to another site where they could access the high-density feeder, and a small number of birds (*N* = 37) moved between control and experimental sites, i.e. experienced control and the high- or low-density treatment).

### Site Level Changes

#### Change in local population density

**Figure 2:**
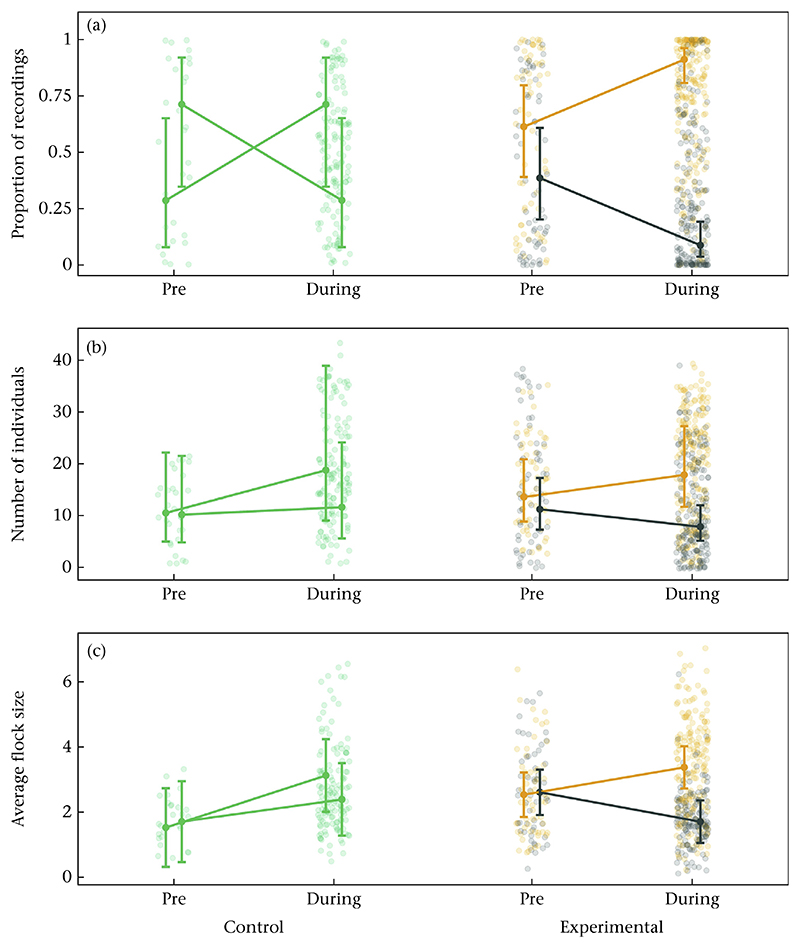
Comparison of (a) the proportion of recordings, (b) the number of individuals and (c) average flock size within and between the high- and low-density feeders before (pre) and during the density manipulation. The raw data and model estimates ± the 95% confidence interval are shown for all sites. Data from the high-density feeder are shown in orange, data from the low-density feeder in grey and data from the control sites in green.

We tested whether local densities, measured as the daily proportion of recordings, the number of visiting individuals and the average flock size changed in relation to our experimental manipulation. All corresponding model results, estimated marginal means and pairwise comparisons can be found in Tables 1, A1–A6. The proportion of recordings at an experimental site increased on average from 61% to 91% between the pre-experimental period and the experimental period at the high-density feeder and decreased from 39% to 9% for the low-density feeder (Fig. 2a, model estimates for all figures were extracted using the ‘effects’ R package; Fox & Weisberg, 2019). Similarly, the number of recorded individuals increased by 32% from the pre-experimental period to the experimental period at the high-density feeder, and decreased by 30% for the low-density feeder (Fig. 2b, Tables A3, A4). Further, average flock size increased by 33% from the pre-experimental period to the experimental period at the high-density feeder, and decreased by 34% for the low-density feeder (Fig. 2c, Tables A5, A6). In the pre-experimental period, low- and high-density feeders did not differ in the proportion of recordings (Table A2), the number of visiting individuals (Table A4) or the average flock size (Table A6). During the experiment, however, feeders differed with on average 91% of recordings at the high-density feeder and only 9% at the low-density feeder (Table A2), and the low-density feeder was visited on average by 56% fewer individuals (Table A4) and 49% smaller flock sizes (Table A6) compared to the high-density feeder. At the two control sites, feeders did not differ in the proportion of recordings, the number of visiting individuals and the average flock size both prior to and during the experiment (Fig. 2a–c, Tables A1–A6). The proportion of recordings, the number of individuals and the average flock size increased at one feeder from the pre-experimental period to the experimental period (Fig. 2a–c, Tables A1–A6) and the proportion of recordings decreased at the corresponding other feeder (Fig. 2a, Tables A1–A6). We detected no effects of experimental day (Tables A1, A3, A6).

#### Change in global social network structure

**Figure 3:**
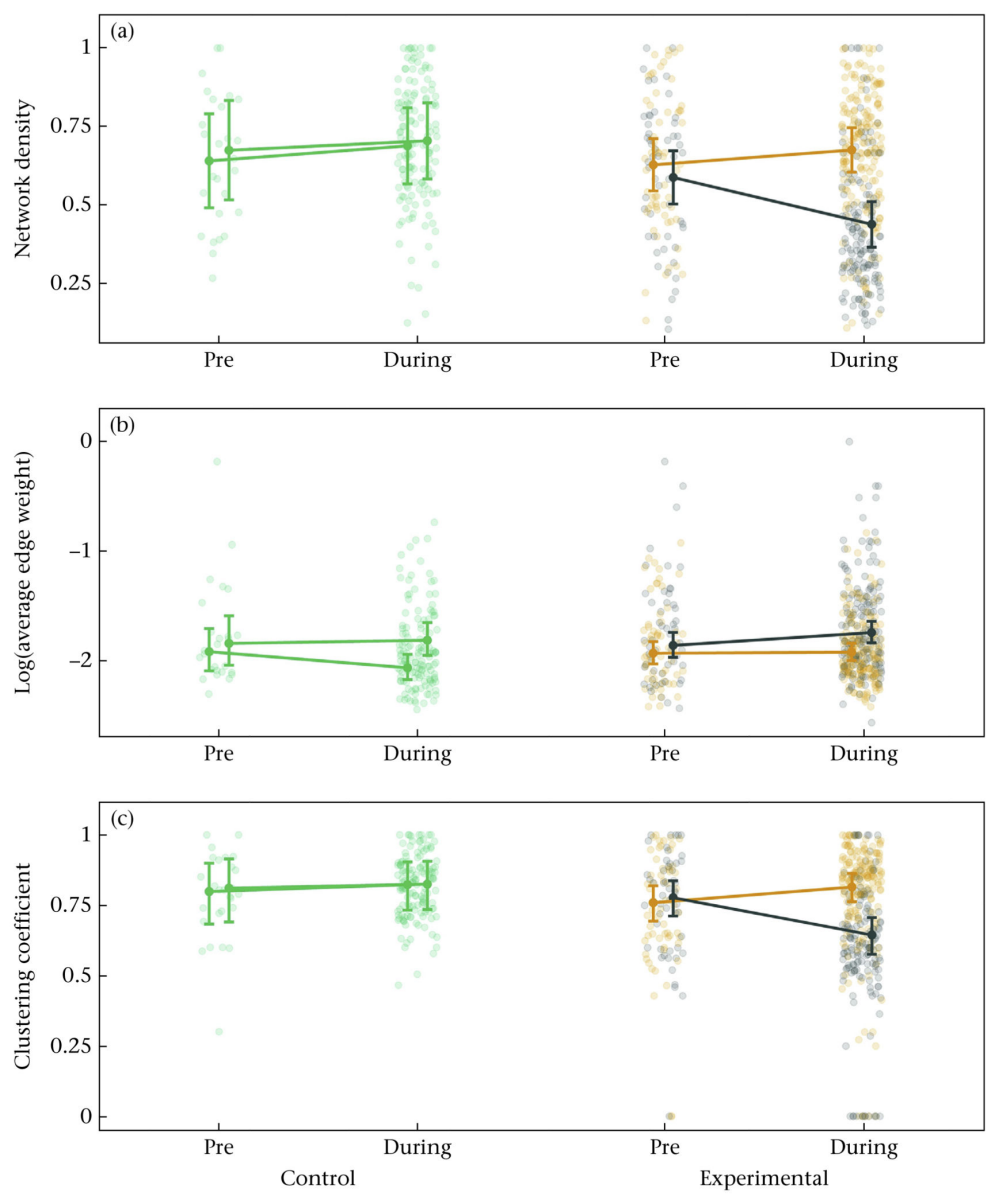
Comparison in global network characteristics within and between the high- and low-density feeders before and during the density manipulation. The raw data and model estimates ± the 95% confidence interval are shown for (a) network density, (b) average edge weight (log-transformed for better visualization) and (c) global clustering coefficient. Data from the high-density feeder are shown in orange, data from the low-density feeder in dark grey and data from the control sites in green.

We tested whether the experimental manipulation of local population density led to changes in the global social network structure. All corresponding model results, estimated marginal means and pairwise comparisons can be found in Tables 1, A7–A12. The network density at the low-density feeders decreased on average by 26% between the pre-experimental period and the experimental period (Fig. 3a, Table A8). Network densities at the high-density feeder remained unchanged (Fig. 3a, Table A8). Average edge weight remained unchanged at both feeders (Fig. 3b, Table A10). The global clustering coefficient at the low-density feeders decreased on average by 31% between the pre-experimental period and the experimental period (Fig. 3c, Table A12). The clustering coefficient at the high-density feeder remained unchanged (Fig. 3c, Table A12). In the pre-experimental period, low- and high-density feeders did not differ in network density (Table A8), the average edge weight (Table A10) and the clustering coefficient (Table A12). During the experiment, feeders of the different treatments differed, with the low-density feeder having on average 35% lower network densities (Table A8), 16% higher average edge weights (Table A10) and 37% lower clustering coefficients (Table A12) compared to the high-density feeder. At the two control sites, feeders did not differ in network density, the average edge weight and clustering both prior to and during the experiment (Fig. 3a–c, Tables A8, A10, A12), except that average edge weight differed between the two control feeders during the ‘experimental’ period (Table A10). Further, each control feeder (i.e. c1 and c2) did not change in network density, the average edge weight and clustering between the pre-experimental period and the experimental period (Fig. 3a–c, Tables A8, A10, A12).

#### Assortment by experimental density assignment

**Figure 4:**
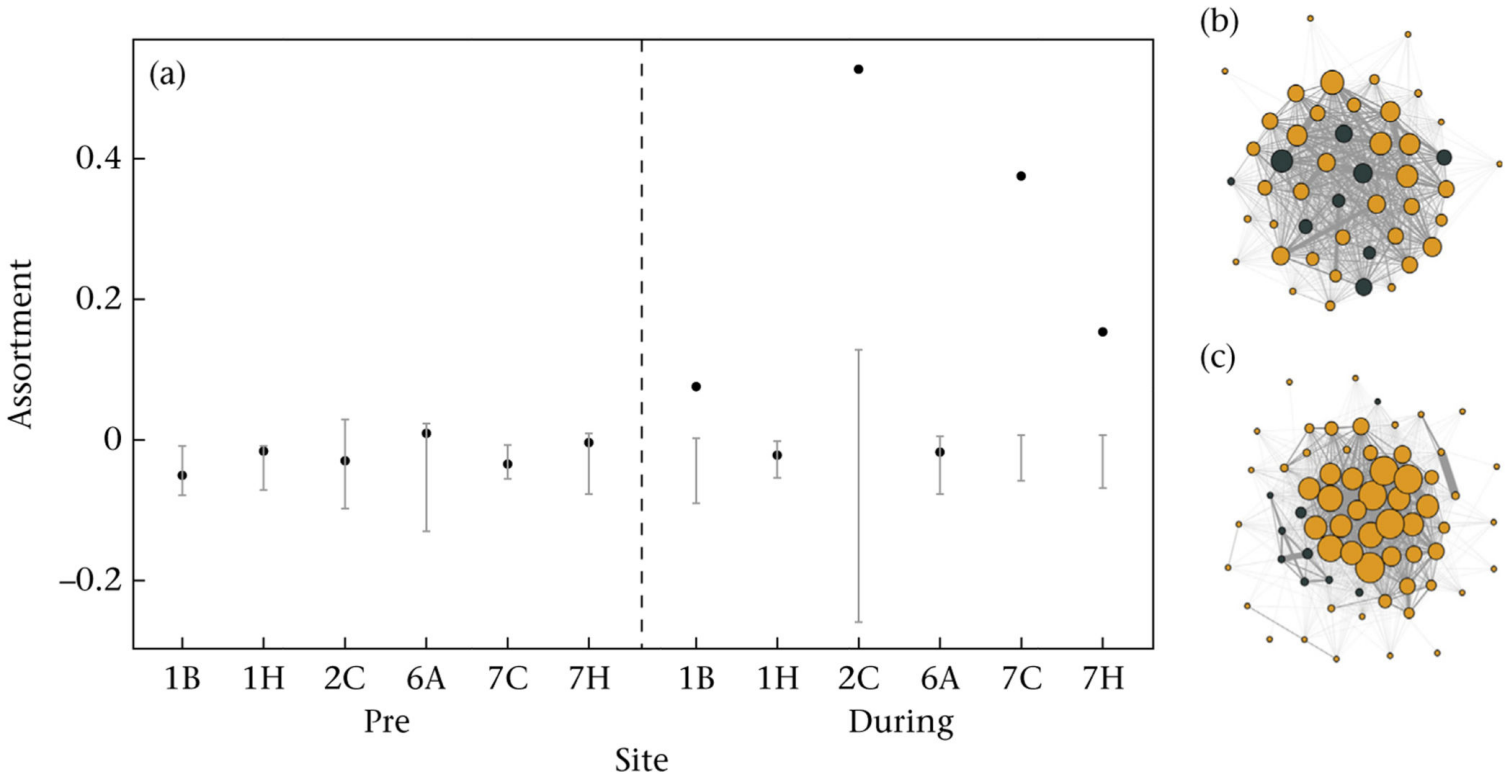
(a) Assortment for the whole network in each period and at each site. Dots show the observed assortativity coefficient and error bars the 95% range of coefficients generated from 1000 random networks. (b, c) Example networks for site 7C with the clearest assortment, (b) prior to and (c) during the manipulation. Colour represents the treatment individuals have been assigned to (orange = high, grey = low), node size represents each individual’s degree and thickness of lines between individuals represents the association strength (i.e. time spent foraging together).

We found no strong evidence for assortment by treatment (i.e. assigned low- or high-density treatment) in the pre-experimental period at all six experimental sites (Newman’s weighted assortativity coefficient ± SE in the pre-experimental period: 1B = –0.05 ± 0.04, 1H = 0.02 ± 0.04, 2C = −0.03 ± 0.04, 6A = 0.01 ± 0.06, 7C = −0.03 ± 0.03, 7H = −0.003 ± 0.04) and values did not differ from the range of assortativity coefficients generated from the permutated data (Fig. 4a–c). However, experimental changes in local population density resulted in changes in the assortment of the network by treatment at four of six sites (Newman’s weighted assortativity coefficient ± SE during the experimental period: 1B = 0.08 ± 0.05, 1H = −0.02 ± 0.03, 2C = 0.53 ± 0.08, 6A = 0.02 ± 0.03, 7C = 0.38 ± 0.04, 7H = −0.15 ± 0.04) and could not be explained by the null model (Fig. 4a). Results on the daily assortativity coefficients can be found in Fig. A3.

### Individual Level Changes

#### Change in visiting activity and average flock size

**Figure 5:**
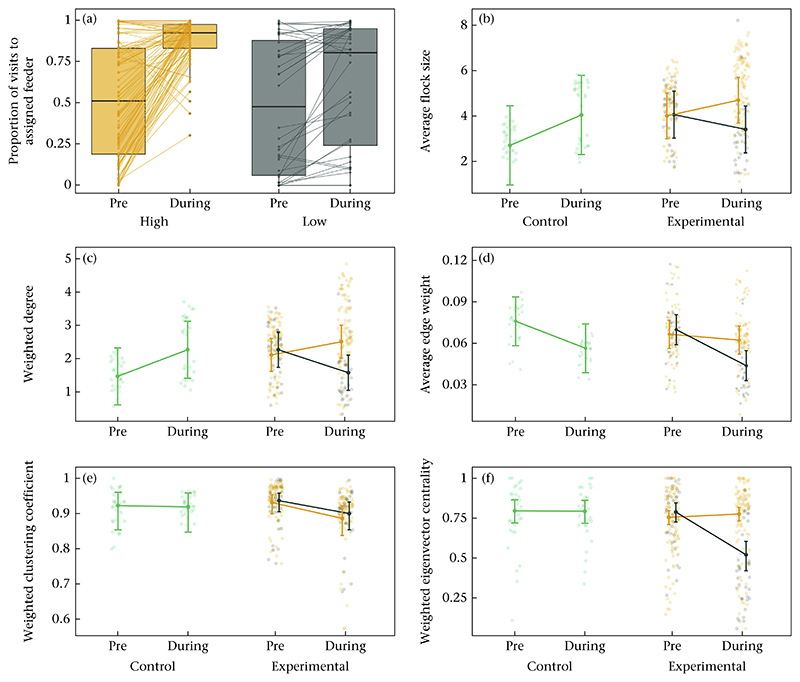
(a) Box plots showing the comparison of the proportion of visits before and during the manipulation to the feeder to which high- (orange) and low-density (grey) individuals had been assigned. Points show each individuals’ proportion prior to and during the experiment connected by a line. The box plots represent the median and 25th and 75th percentiles and the whiskers indicate the values within 1.5 times the interquartile range. (b) Change in average flock size, (c) weighted degree, (d) average edge weight, (e) weighted clustering coefficient, and (f) weighted eigenvector centrality between birds assigned to the high- (orange) and low-density (grey) treatment, and birds at the control sites (green), before and during the density manipulation. The raw data and model estimates ± the 95% confidence interval are shown.

We tested whether individuals changed their visiting patterns in reponse to the imposed density manipulation, that is whether individuals allowed access at the low-density feeder also increased visits to the low-density feeder and vice versa. All corresponding model results, estimated marginal means and pairwise comparisons can be found in Tables A13–A16. To do so we investigated the proportion of visits to the feeder to which great tits had been assigned in the pre-experimental and experimental phase. We found that both low- and high-density individuals increased their visits by on average 13% and 24%, respectively, to the feeder they have been assigned to (Fig. 5a, Tables A13, A14). In addition, high-density birds experienced on average an increase in average flock size by 17% between pre-experimental and experimental period (Fig. 5b, Tables A15, A16). Birds in the low-density treatment experienced smaller flock sizes (by 16%; Fig. 5b, Tables A15, A16). Individuals assigned to the low- and high-density treatments did not differ in mean flock size prior to the density manipulation (Fig. 5b, Table A16). However, during the experiment, birds in the low-density treatment experienced on average 28% smaller flock sizes than birds in the high-density treatment (Fig. 5b, Table A16). At the two control sites, the average flock sizes experienced by individuals increased on average by 50% (Fig. 5b, Table A16).

#### Change in social network position

We tested whether the experimental assignment of individuals to either the low- or high-density treatment led to changes in social network position. All corresponding model results, estimated marginal means and pairwise comparisons, can be found in Tables A17–A24. The weighted degree of individuals assigned to the high-density treatment increased on average by 19% between the pre-experimental period and the experimental period (Fig. 5c, Tables A17, A18). For individuals assigned to the low-density treatment, weighted degree decreased on average by 30% (Fig. 5c, Tables A17, A18). Average edge weight decreased for individuals assigned to the low-density treatment by 37% between the pre-experimental period and the experimental period (Fig. 5d, Tables A19, A20), whereas average edge weight remained unchanged for individuals in the high-density treatment (Fig. 5d, Tables A19, A20). The weighted clustering coefficient of individuals both decreased on average by 5% and 4% for birds assigned to the high- and low-density treatment, respectively (Fig. 5e, Tables A21, A22). The weighted eigenvector centrality decreased for individuals assigned to the low-density by on average 57% between the pre-experimental period and the experimental period (Fig. 5f, Tables A23, A24). Eigenvector centrality remained unchanged for individuals in the high-density treatment (Fig. 5f, Tables A23, A24). In the pre-experimental period, individuals of the low- and high-density treatments did not differ in weighted degree (Fig. 5c, Table A18), the average edge weight (Fig. 5d, Table A20), weighted clustering coefficient (Fig. 5e, Table A22) and weighted eigenvector centrality (Fig. 5f, Table A24). During the experiment, individuals differed with the low-density individuals having on average 37% lower weighted degrees (Fig. 5c, Table A18), 30% lower average edge weights (Fig. 5d, Table A20) and 55% lower eigenvector centrality (Fig. 5f, Table A24) compared to the high-density individuals. Individuals of the different treatments did not differ in weighted clustering coefficient (Fig. 5e, Table A22). At the two control sites, individuals on average increased weighted degree by 54% (Fig. 5c, Table A18). Average edge weight, weighted clustering coefficient and weighted eigenvector centrality did not differ between the two periods (Fig. 5d–f; Tables A20, A22, A24).

#### Patch Discovery Experiment

**Figure 6:**
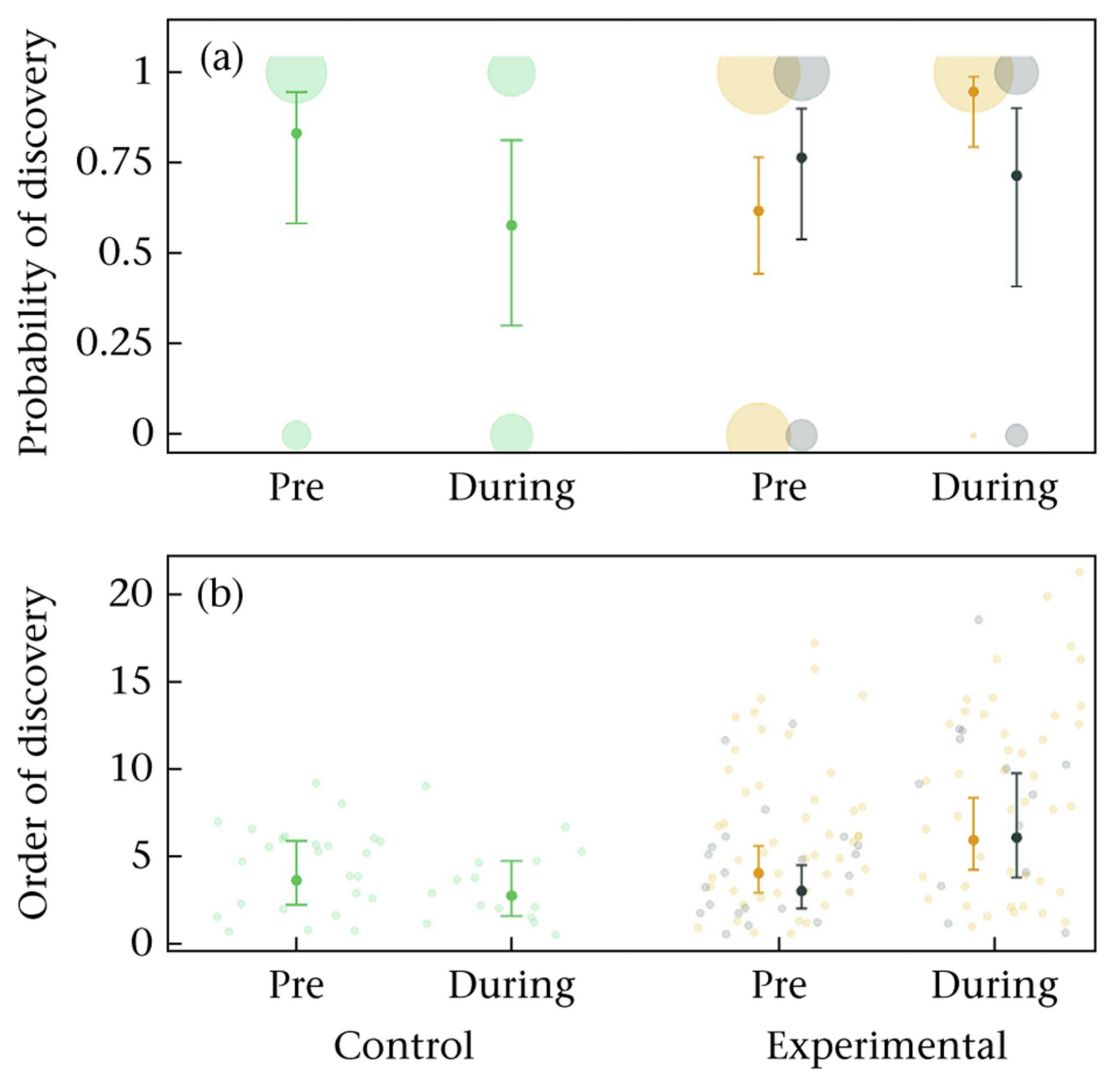
(a) Probability and (b) order of discovery in relation to an individual’s treatment (green = control, orange = high density, grey = low density). The raw data and model estimates ± the 95% confidence interval are shown.

From the 148 great tits selected for the analysis (see Methods), 119 discovered a novel feeder. In total, we recorded 164 discovery events by the 119 great tits of which 90 were made by an individual assigned to the high-density treatment, 34 by individuals in the low-density treatment and 40 by individuals in the control sites. From the 119 individuals, five discovered a novel patch at two different experimental sites. However, we found no evidence that birds assigned to the high-density treatment were either more likely to discover a novel feeder or were faster to do so compared to birds assigned to the low-density treatment (Fig. 6, Tables A25–A30). Both prior to and during the experimental manipulation, birds in the high-density treatment and birds in the low-density treatment did not differ in the probability (estimated mean probability to discover: pre high = 0.62, pre low = 0.77; during high = 0.95, during low = 0.72; Tables A25, A26) or speed of discovering a novel food resource (estimated mean order of discovery: pre high = 4.14, pre low 3.12; during high = 6.04, during low = 6.18; Tables A27, A28; estimated mean latency of discovery: pre high 270, pre low 215; during high 268, during low 300; Tables A29, A30). However, during the experimental period birds assigned to the high-density treatment had an increased likelihood of discovering the novel food source (estimated mean probability of discovering: pre high: 0.62, during high: 0.95; Tables A25, A26). At the two control sites, the probability or speed of discovering the novel feeder did not differ between the two periods (Tables A25–A30).

## Discussion

Here, we demonstrated how automated feeding stations can be used to experimentally manipulate local population density and social structure in wild great tits. Our experiments show that locally increased densities led to denser and more clustered local social structures. On the individual level, birds assigned to the low-density treatment foraged on average in smaller flocks, exhibited fewer and weaker social connections and occupied less central network positions compared to birds assigned to the high-density treatment. However, contrary to our predictions and common assumptions, we found no evidence that the density manipulation influenced individuals’ likelihood and speed of discovering novel food patches.

Using automated feeding stations, we assigned great tits to low- and high-density treatments, and successfully created low- and high-density locations that differed in the number of individuals, the proportion of visits and the average group sizes recorded (Fig. 2). Birds assorted by their assigned treatment at most experimental sites (i.e. stronger association strengths between individuals of the same treatment; Fig. 4) and increased their foraging visits to the feeder to which they had been assigned, whereby birds in the high-density treatment almost exclusively visited the high-density feeder during the experimental period (Fig. 5a). Individuals assigned to the low-density treatment still visited the high-density feeder to an appreciable extent (Fig. 5a).

In addition, we showed that the experimental treatment affected individuals’ social environment. While the average flock size experienced by individuals at the control sites increased to a similar extent as at the high-density feeders (Fig. 5b), at experimental sites, birds in the low-density treatment experienced on average smaller foraging flocks than birds in the high-density treatment (Fig. 5b). Further, birds in the low-density treatment had fewer social connections, and less central network positions compared to birds assigned to the high-density treatment (Fig. 5c, f). However, the observed differences in the social environment between individuals of the low- and high-density treatment were relatively small in some cases. For instance, birds in the high-density treatment foraged on average in flocks of 4.7 individuals whereas birds in the low-density treatment foraged on average with 3.4 individuals (Table A16, Fig. 5b). This is presumably because the density manipulation was in place for a relatively short time (about 6 weeks) and previous work is suggesting that imposing segregation for longer increases the amount of segregation over time (Firth & Sheldon, 2015). Further, our experimental manipulation split past and future breeding pairs that are known to continue foraging together, even though they are not able to access the same resources (Firth et al., 2015). Specifically, 42% of the birds in the low-density treatment had been assigned to a different treatment than their past or future breeding partner, while only 8% of the birds in the high-density treatment experienced a potential separation. Note, however, that we cannot fully distinguish whether previous or future breeding partners had really been separated by our experimental manipulation. This is because previous partners may have already divorced and future partners may not have yet bonded. In addition, great tits may prefer to forage in larger social groups because, for instance, they offer protection against predators. Thus, birds in the low-density treatment may have spent more time at the ‘incorrect’ feeder (i.e. the high-density feeder; see Fig. 5a) to continue foraging with their specific desired future breeding partner and also to remain foraging in larger flocks. Further research could now assess whether the overall observed small differences in the social environment between individuals in the low- and high-density treatments may thus be caused by birds in the low-density treatment actively attempting to compensate for their experienced greater social disruption, particularly as previous studies on this species have suggested such social compensation (Firth et al., 2015, 2017).

Great tits use social information across a variety of foraging contexts (Aplin et al., 2012; Farine et al., 2015; Firth et al., 2016) and birds occupying more central social network positions have been demonstrated to be more likely to discover novel food patches (Aplin et al., 2012). We found no evidence that our experimental density manipulation affected an individual’s likelihood and speed of discovering novel food (Fig. 6). Even though the density manipulation led to, on average, more central social network positions of individuals assigned to the high-density treatment (i.e. higher weighted degree and eigenvector centrality, Fig. 5c, f), they were not more likely, or faster, to discover novel food patches. This may be because changes in individual sociality induced by the density manipulation were relatively small (see above) and thus did not greatly impact an individual’s social network position and thereby access to information. Future studies using such experimental density manipulations over a prolonged period of time (e.g. over the whole winter period) may allow for a better establishment of the predicted differences in local density and particularly individual social environments. Further, the novel food patches may have been too close (40 m) to the known feeders and thus social information transmission may have not been necessary for discovering the novel patches. Therefore, studies providing novel food resources that are more difficult to find or using other experimental approaches to test differences in information transmission (e.g. puzzle boxes; Aplin et al., 2015; Kendal et al., 2009) in relation to variation in population density may provide different results. In addition, it is possible that phenotypic traits, which we did not consider in our experimental manipulation, influenced the likelihood and speed of patch discovery. While a previous study found no influence of age and sex on patch discovery in tits over-and-above the effect of social learning (Aplin et al., 2012), it is possible that other traits such as personality (i.e. exploration behaviour) could impact patch discovery and may have thus limited our ability to detect effects of our experimental manipulation. Finally, a limitation of our study is that we could only capture the social associations at the feeder and not elsewhere. It is possible that due to the experimental restrictions, birds (specifically those in the low-density treatment) increasingly foraged and associated with conspecifics away from the feeder, limiting our ability to detect the full range of social associations even though previous work has shown that associations at the feeders carry over and represent associations in other contexts (Firth & Sheldon, 2015, 2016).

We demonstrated that the experimental manipulation of local population density led to differences in the local social structure, whereby lower densities led to, on average, smaller group sizes, and less dense and clustered local social networks (Figs. 2 and 3). Several studies demonstrate a positive relationship between population density, encounter probability and group size in species of unstable groups (Gerard et al., 2002; Lawes & Nanni, 1993; Pépin & Gerard, 2008; Southwell, 1984). For instance, in Pyrenean chamois, *Rupicapra pyrenaica*, mean group size and the rate at which groups joined and split increased with increasing population density (Pépin & Gerard, 2008). The social structure can have profound ecological and evolutionary implications (Evans et al., 2021; McDonald & Pizzari, 2018; Montiglio et al., 2018; Sah et al., 2018). Hence, generating a better understanding of the underlying mechanisms that shape variation in population density would be crucial to uncover its impact on social structure and subsequent population dynamics. While we experimentally induced variation in local population density, density also naturally varies across different spatiotemporal scales within and across populations (Jacquier et al., 2021; Wilkin et al., 2006). For example, features of the spatial environment such as different habitat characteristics (e.g. vegetation and the distribution of resources), predation and demographic processes (emigration and immigration) can influence population density and may subsequently impact social structure and population processes (Borgeaud et al., 2017; He et al., 2021; Lawes & Nanni, 1993; Shizuka & Johnson, 2020; Webber et al., 2023).

In our study, we investigated the effect of the experimental density manipulation on the probability and speed of information acquisition of individuals. However, research comparing information transmission in relation to population density on a local level, rather than on the individual level, warrants further investigation. For instance, comparing the efficiency of spread (e.g. the time until the majority of individuals in a group are knowledgeable) between subpopulations of varying densities (with no or little movement between subpopulations) may reveal an increased transmission efficiency at high-density locations, similarly to findings on disease transmission (Ryder et al., 2005; Storm et al., 2013). Examining the potential density dependence of information transmission is crucial given the natural spatiotemporal variation in population density (Jacquier et al., 2021; Wilkin et al., 2006), and may thus have important consequences for our understanding of when and where novel behaviours are likely to spread (Somveille et al., 2018). For instance, at low-density locations novel behaviours may not spread successfully even though the behaviour may be advantageous. Therefore, studying how spatiotemporal variation in ecological features shapes local social structure and information transmission would be crucial to better understand whether and how behaviours spread, and subsequently the establishment of local traditions and cultures.

In contrast to disease transmission, individuals can actively decide how to act upon novel information (e.g. whether to adopt a novel behaviour or not). Therefore, studying the density dependence of information transmission may not necessarily follow the same dynamics as disease transmission. For instance, across taxa, individuals often only adopt a new behaviour once the majority of social associates performs the behaviour (i.e. conformist learning; Aplin et al., 2015; Danchin et al., 2018; Pike & Laland, 2010; Van de Waal et al., 2013). In such a case, individuals with more social connections are expected to adopt a novel behaviour later than individuals with fewer social connections (Firth, 2020; Firth et al., 2020). In fact, contemporary research is suggesting that in natural systems, the correlation between individual social network position and information acquisition may be dependent on the social learning rule at play (Beck et al., 2023). Future studies exploring the density dependence of information transmission using experimental approaches like the ones outlined here could advance the field further through consideration of how different social learning mechanisms may shape behavioural spread.

## Supplementary Material

Appendix

## Figures and Tables

**Table 1:** Summary of the main effects comparing the low- and high-density treatments

Low density	High density
**Local level**	
Fewer recordings	More recordings
Fewer individuals	More individuals
Smaller flock sizes	Larger flock sizes
Lower network density	Higher network density
Lower clustering coefficient	Higher clustering coefficient
**Individual level**	
Smaller flock sizes	Larger flock sizes
Lower weighted degree	Higher weighted degree
Lower average edge weight	Higher average edge weight
Lower eigenvector centrality	Higher eigenvector centrality

The main effects for the low- and high-density location (local level) and individuals assigned to the low- and high-density treatment (individual level) during the experimental density manipulation.

## Data Availability

Analyses and figures reported in this article can be reproduced using the data and code provided at the Open Science Framework (OSF): https://doi.org/10.17605/OSF.IO/FGJ94.

## References

[R1] Albery G (2022). Density dependence and disease dynamics: Moving towards a predictive framework. EcoEvoRxiv.

[R2] Anderson RM (1982). Theoretical basis for the use of pathogens as biological control agents of pest species. Parasitology.

[R3] Aplin LM, Farine DR, Morand-Ferron J, Cockburn A, Thornton A, Sheldon BC (2015). Experimentally induced innovations lead to persistent culture via conformity in wild birds. Nature.

[R4] Aplin LM, Farine DR, Morand-Ferron J, Sheldon BC (2012). Social networks predict patch discovery in a wild population of songbirds. Proceedings of the Royal Society B: Biological Sciences.

[R5] Atton N, Galef BJ, Hoppitt W, Webster MM, Laland KN (2014). Familiarity affects social network structure and discovery of prey patch locations in foraging stickleback shoals. Proceedings of the Royal Society B: Biological Sciences.

[R6] Beck KB, Sheldon BC, Firth JA (2023). Social learning mechanisms shape transmission pathways through replicate local social networks of wild birds. eLife.

[R7] Borgeaud C, Sosa S, Sueur C, Bshary R (2017). The influence of demographic variation on social network stability in wild vervet monkeys. Animal Behaviour.

[R8] Brunner JL, Beaty L, Guitard A, Russell D (2017). Heterogeneities in the infection process drive ranavirus transmission. Ecology.

[R9] Buchanan K, Burt de Perera T, Carere C, Carter T, Hailey A, Hubrecht R, Jennings D, Metcalfe N, Pitcher T, Peron F (2012). Guidelines for the treatment of animals in behavioural research and teaching. Animal Behaviour.

[R10] Cairns SJ, Schwager SJ (1987). A comparison of association indices. Animal Behaviour.

[R11] Colman E, Colizza V, Hanks EM, Hughes DP, Bansal S (2021). Social fluidity mobilizes contagion in human and animal populations. eLife.

[R12] Csardi G, Nepusz T (2006). The igraph software package for complex network research. InterJournal, Complex Systems.

[R13] Culina A, Hinde CA, Sheldon BC (2015). Carry-over effects of the social environment on future divorce probability in a wild bird population. Proceedings of the Royal Society B: Biological Sciences.

[R14] Danchin E, Nöbel S, Pocheville A, Dagaeff AC, Demay L, Alphand M, Ranty-Roby S, Van Renssen L, Monier M, Gazagne E (2018). Cultural flies: Conformist social learning in fruitflies predicts long-lasting mate-choice traditions. Science.

[R15] Ekman J (1989). Ecology of non-breeding social systems of Parus. Wilson Bulletin.

[R16] Evans JC, Hodgson DJ, Boogert NJ, Silk MJ (2021). Group size and modularity interact to shape the spread of infection and information through animal societies. Behavioral Ecology and Sociobiology.

[R17] Farine DR (2013). Animal social network inference and permutations for ecologists in R using asnipe. Methods in Ecology and Evolution.

[R18] Farine DR (2014). Measuring phenotypic assortment in animal social networks: Weighted associations are more robust than binary edges. Animal Behaviour.

[R19] Farine DR, Aplin LM, Sheldon BC, Hoppitt W (2015). Interspecific social networks promote information transmission in wild songbirds. Proceedings of the Royal Society B: Biological Sciences.

[R20] Firth JA (2020). Considering complexity: Animal social networks and behavioural contagions. Trends in Ecology & Evolution.

[R21] Firth JA, Albery GF, Beck KB, Jarić I, Spurgin LG, Sheldon BC, Hoppitt W (2020). Analysing the social spread of behaviour: Integrating complex contagions into network based diffusions. ArXiv Preprint.

[R22] Firth JA, Sheldon BC (2015). Experimental manipulation of avian social structure reveals segregation is carried over across contexts. Proceedings of the Royal Society B: Biological Sciences.

[R23] Firth JA, Sheldon BC (2016). Social carry-over effects underpin trans-seasonally linked structure in a wild bird population. Ecology Letters.

[R24] Firth JA, Sheldon BC, Farine DR (2016). Pathways of information transmission among wild songbirds follow experimentally imposed changes in social foraging structure. Biology Letters.

[R25] Firth JA, Voelkl B, Crates RA, Aplin LM, Biro D, Croft DP, Sheldon BC (2017). Wild birds respond to flockmate loss by increasing their social network associations to others. Proceedings of the Royal Society B: Biological Sciences.

[R26] Firth JA, Voelkl B, Farine DR, Sheldon BC (2015). Experimental evidence that social relationships determine individual foraging behavior. Current Biology.

[R27] Fox J, Weisberg S (2019). An R companion to applied regression.

[R28] Gerard JF, Bideau E, Maublanc ML, Loisel P, Marchal C (2002). Herd size in large herbivores: Encoded in the individual or emergent?. Biological Bulletin.

[R29] Hasenjager MJ, Leadbeater E, Hoppitt W (2021). Detecting and quantifying social transmission using network-based diffusion analysis. Journal of Animal Ecology.

[R30] Hein AM, McKinley SA (2013). Sensory information and encounter rates of interacting species. PLoS Computational Biology.

[R31] He P, Montiglio PO, Somveille M, Cantor M, Farine DR (2021). The role of habitat configuration in shaping animal population processes: A framework to generate quantitative predictions. Oecologia.

[R32] Hopkins SR, Fleming-Davies AE, Belden LK, Wojdak JM (2020). Systematic review of modelling assumptions and empirical evidence: Does parasite transmission increase nonlinearly with host density?. Methods in Ecology and Evolution.

[R33] Hoppitt W, Laland KN (2013). Social learning.

[R34] Hu H, Nigmatulina K, Eckhoff P (2013). The scaling of contact rates with population density for the infectious disease models. Mathematical Biosciences.

[R35] Hutchinson JMC, Waser PM (2007). Use, misuse and extensions of ‘ideal gas’ models of animal encounter. Biological Reviews.

[R36] Jacquier M, Vandel JM, Léger F, Duhayer J, Pardonnet S, Say L, Devillard S, Ruette S (2021). Breaking down population density into different components to better understand its spatial variation. BMC Ecology and Evolution.

[R37] Jirotkul M (1999). Population density influences male–male competition in guppies. Animal Behaviour.

[R38] Kendal RL, Kendal JR, Hoppitt W, Laland KN (2009). Identifying social learning in animal populations: A new ‘option-bias’ method. PLoS One.

[R39] Kokko H, Rankin DJ (2006). Lonely hearts or sex in the city? Density-dependent effects in mating systems. Philosophical Transactions of the Royal Society B: Biological Sciences.

[R40] Kulahci IG, Rubenstein DI, Bugnyar T, Hoppitt W, Mikus N, Schwab C (2016). Social networks predict selective observation and information spread in ravens. Royal Society Open Science.

[R41] Lawes MJ, Nanni RF (1993). The density, habitat use and social organisation of Dorcas Gazelles (Gazella dorcas) in Makhtesh Ramon, Negev Desert, Israel. Journal of Arid Environments.

[R42] Lenth R, Singmann H, Love J, Buerkner P, Herve M (2019). Emmeans: Estimated marginal means, aka least-squares means.

[R43] Magnusson A, Skaug H, Nielsen A, Berg C, Kristensen K, Maechler M, Van Bentham K, Bolker B, Brooks M (2017). glmmTMB: Generalized linear mixed models using template model builder. R package version 01 3.

[R44] McDonald GC, Pizzari T (2018). Structure of sexual networks determines the operation of sexual selection. Proceedings of the National Academy of Sciences.

[R45] Montiglio P, McGlothlin JW, Farine DR (2018). Social structure modulates the evolutionary consequences of social plasticity: A social network perspective on interacting phenotypes. Ecology and Evolution.

[R46] Newman MEJ (2003). Mixing patterns in networks. Physical Review E.

[R47] Pasquaretta C, Levé M, Claidiere N, Van de Waal E, Whiten A, MacIntosh AJJ, Pelé M, Bergstrom ML, Borgeaud C, Brosnan SF (2014). Social networks in primates: Smart and tolerant species have more efficient networks. Scientific Reports.

[R48] Pépin D, Gerard JF (2008). Group dynamics and local population density dependence of group size in the Pyrenean chamois, Rupicapra pyrenaica. Animal Behaviour.

[R49] Pike TW, Laland KN (2010). Conformist learning in nine-spined sticklebacks’ foraging decisions. Biology Letters.

[R50] Pitcher TJ, Magurran AE, Winfield IJ (1982). Fish in larger shoals find food faster. Behavioral Ecology and Sociobiology.

[R51] Psorakis I, Roberts SJ, Rezek I, Sheldon BC (2012). Inferring social network structure in ecological systems from spatio-temporal data streams. Journal of the Royal Society Interface.

[R52] R Core Team (2020). R Foundation for Statistical Computing.

[R53] Regan CE, Beck KB, McMahon K, Crofts S, Firth JA, Sheldon BC (2022). Social phenotype-dependent selection of social environment in wild great and blue tits: An experimental study. Proceedings of the Royal Society B: Biological Sciences.

[R54] Ryder JJ, Webberley KM, Boots M, Knell RJ (2005). Measuring the transmission dynamics of a sexually transmitted disease. Proceedings of the National Academy of Sciences.

[R55] Sah P, Mann J, Bansal S (2018). Disease implications of animal social network structure: A synthesis across social systems. Journal of Animal Ecology.

[R56] Sanchez JN, Hudgens BR (2015). Interactions between density, home range behaviors, and contact rates in the Channel Island fox (*Urocyon littoralis*). Ecology and Evolution.

[R57] Savill P, Perrins C, Kirby K, Fisher N (2011). Wytham Woods: Oxford’s ecological laboratory.

[R58] Schakner ZA, Petelle MB, Tennis MJ, Van der Leeuw BK, Stansell RT, Blumstein DT (2017). Social associations between California sea lions influence the use of a novel foraging ground. Royal Society Open Science.

[R59] Shizuka D, Johnson AE (2020). How demographic processes shape animal social networks. Behavioral Ecology.

[R60] Snijders L, Krause S, Tump AN, Breuker M, Ortiz C, Rizzi S, Ramnarine IW, Krause J, Kurvers RHJM (2021). Causal evidence for the adaptive benefits of social foraging in the wild. Communications Biology.

[R61] Somveille M, Firth JA, Aplin LM, Farine DR, Sheldon BC, Thompson RN (2018). Movement and conformity interact to establish local behavioural traditions in animal populations. PLoS Computational Biology.

[R62] Southwell CJ (1984). Variability in grouping in the eastern grey kangaroo, *Macropus giganteus I*. Group density and group size. Wildlife Research.

[R63] Storm DJ, Samuel MD, Rolley RE, Shelton P, Keuler NS, Richards BJ, Van Deelen TR (2013). Deer density and disease prevalence influence transmission of chronic wasting disease in white-tailed deer. Ecosphere.

[R64] Vander Wal E, Laforge MP, McLoughlin PD (2014). Density dependence in social behaviour: Home range overlap and density interacts to affect conspecific encounter rates in a gregarious ungulate. Behavioral Ecology and Sociobiology.

[R65] Vander Wal E, Van Beest FM, Brook RK (2013). Density-dependent effects on group size are sex-specific in a gregarious ungulate. PLoS One.

[R66] Van de Waal E, Borgeaud C, Whiten A (2013). Potent social learning and conformity shape a wild primatés foraging decisions. Science.

[R67] Webber Q, Albery G, Farine DR, Pinter-Wollman N, Sharma N, Spiegel O, Vander Wal E, Manlove K (2023). Behavioural ecology at the spatial-social interface. Biological Reviews.

[R68] Whitehead H (2008). Analyzing animal societies: Quantitative methods for vertebrate social analysis.

[R69] Whiten A (2021). The burgeoning reach of animal culture. Science.

[R70] Wilkin TA, Garant D, Gosler AG, Sheldon BC (2006). Density effects on life-history traits in a wild population of the great tit *Parus major*: Analyses of long-term data with GIS techniques. Journal of Animal Ecology.

