# Supplementary material for: Experimental manipulation of population density in a wild bird alters social structure but not patch discovery rate
Source: Anim Behav. Author manuscript; Available in PMC 2025 Oct 13. (PMC7618250; doi:10.1016/j.anbehav.2023.12.010)
Supplement: Appendix [file EMS209389-suppement-Appendix.pdf]

Appendix

Table A1
Results of the model examining the effects of the experimental density manipulation on the proportion of recordings

	Estimate	SE	z	*P*
Intercept	−0.911	0.891	−1.022	0.307
Period during	1.820	0.035	51.752	<0.0001
Condition c2	1.821	1.107	1.646	0.100
Condition high	1.374	0.908	1.513	0.120
Condition low	0.448	0.908	0.493	0.622
Experimental day	0.000	0.020	0.000	1.000
Period during:condition c2	−3.639	0.050	−73.186	<0.0001
Period during:condition high	0.056	0.039	1.441	0.150
Period during:condition low	−3.695	0.039	−94.762	<0.0001

Period refers to the experimental period: prior versus during the density manipulation. Condition refers to the different experimental treatments: low = low density, high = high density; c1 and c2 = control feeders. Experimental day describes the day within each experimental period.

Table A3
Results of the model examining the effects of the experimental density manipulation on the number of visiting individuals

	Estimate	SE	*z*	*P*
Intercept	2.423	0.397	6.101	<0.0001
Period during	0.581	0.1	5.806	<0.0001
Condition c2	−0.03	0.452	−0.067	0.946
Condition high	0.257	0.44	0.585	0.559
Condition low	0.066	0.441	0.149	0.882
Experimental day	−0.004	0.005	−0.735	0.462
Period during:condition c2	−0.45	0.14	−3.218	0.001
Period during:condition high	−0.307	0.11	−2.78	0.005
Period during:condition low	−0.94	0.112	−8.386	<0.0001

Period refers to the experimental period: prior versus during the density manipulation. Condition refers to the different experimental treatments: low = low density, high = high density; c1 and c2 = control feeders. Experimental day describes the day within each experimental period.

Table A2
Estimated marginal means and pairwise comparisons of the proportion of recordings

Period	Condition	Probability	SE	Lower CL	Upper CL
**Estimated marginal means**					
Pre	c1	0.287	0.160	0.079	0.652
During	c1	0.713	0.161	0.347	0.920
Pre	c2	0.713	0.160	0.348	0.920
During	c2	0.287	0.161	0.080	0.652
Pre	High	0.614	0.110	0.391	0.797
During	High	0.912	0.037	0.808	0.962
Pre	Low	0.386	0.110	0.203	0.609
During	Low	0.088	0.037	0.038	0.193
**Pairwise comparisons**					
Comparison	Odds ratio	SE	*t*	*P*	
Pre c1/during c1	0.162	0.006	−51.752	<0.0001	
Pre c1/pre c2	0.162	0.179	−1.646	0.722	
Pre c1/during c2	0.998	1.104	−0.002	1.000	
Pre c1/pre high	0.253	0.230	−1.513	0.801	
Pre c1/during high	0.039	0.035	−3.578	0.009	
Pre c1/pre low	0.639	0.580	−0.493	1.000	
Pre c1/during low	4.170	3.785	1.573	0.766	
During c1/pre c2	0.998	1.104	−0.002	1.000	
During c1/during c2	6.158	6.811	1.643	0.724	
During c1/pre high	1.562	1.418	0.491	1.000	
During c1/during high	0.239	0.217	−1.575	0.765	
During c1/pre low	3.942	3.577	1.512	0.801	
During c1/during low	25.723	23.337	3.579	0.009	
Pre c2/during c2	6.169	0.217	51.752	<0.0001	
Pre c2/pre high	1.565	1.421	0.493	1.000	
Pre c2/during high	0.240	0.218	−1.573	0.766	
Pre c2/pre low	3.949	3.586	1.513	0.801	
Pre c2/during low	25.771	23.401	3.578	0.009	
During c2/pre high	0.254	0.230	−1.512	0.801	
During c2/during high	0.039	0.035	−3.579	0.009	
During c2/pre low	0.640	0.581	−0.491	1.000	
During c2/during low	4.178	3.791	1.575	0.765	
Pre high/during high	0.153	0.003	−111.216	<0.0001	
Pre high/pre low	2.524	1.643	1.422	0.847	
Pre high/during low	16.468	10.722	4.303	0.001	
During high/pre low	16.468	10.722	4.303	0.001	
During high/during low	107.462	69.953	7.185	<0.0001	
Pre low/during low	6.525	0.110	111.216	<0.0001	

Estimated marginal means and pairwise comparison are presented across all combinations of experimental period (pre, during) and treatment (condition: low, high; control feeder 1 (c1), control feeder 2 (c2)). Shown are the estimated means, standard errors (SE), 95% confidence intervals (CL) for the estimated means, test statistic and the *P* value for each pairwise comparison.

Table A4
Estimated marginal means and pairwise comparisons of the number of individuals visiting

Period	Condition	Rate	SE	Lower CL	Upper CL
**Estimated marginal means**					
Pre	c1	10.485	4.002	4.956	22.183
During	c1	18.752	6.989	9.020	38.983
Pre	c2	10.171	3.889	4.801	21.548
During	c2	11.596	4.337	5.564	24.169
Pre	High	13.562	2.973	8.818	20.857
During	High	17.846	3.863	11.667	27.298
Pre	Low	11.196	2.470	7.260	17.267
During	Low	7.822	1.706	5.098	12.004
**Pairwise comparisons**					
Comparison	Ratio	SE	*t*	*P*	
Pre c1/during c1	0.559	0.056	−5.806	<0.0001	
Pre c1/pre c2	1.031	0.466	0.067	1.000	
Pre c1/during c2	0.904	0.402	−0.226	1.000	
Pre c1/pre high	0.773	0.340	−0.585	0.999	
Pre c1/during high	0.588	0.258	−1.212	0.928	
Pre c1/pre low	0.936	0.413	−0.149	1.000	
Pre c1/during low	1.340	0.589	0.667	0.998	
During c1/pre c2	1.844	0.820	1.376	0.868	
During c1/during c2	1.617	0.707	1.099	0.957	
During c1/pre high	1.383	0.598	0.749	0.995	
During c1/during high	1.051	0.453	0.115	1.000	
During c1/pre low	1.675	0.725	1.191	0.934	
During c1/during low	2.397	1.034	2.026	0.465	
Pre c2/during c2	0.877	0.088	−1.311	0.895	
Pre c2/pre high	0.750	0.331	−0.653	0.998	
Pre c2/during high	0.570	0.250	−1.280	0.906	
Pre c2/pre low	0.908	0.401	−0.218	1.000	
Pre c2/during low	1.300	0.572	0.597	0.999	
During c2/pre high	0.855	0.371	−0.361	1.000	
During c2/during high	0.650	0.281	−0.998	0.975	
During c2/pre low	1.036	0.449	0.081	1.000	
During c2/during low	1.482	0.641	0.910	0.985	
Pre high/during high	0.760	0.035	−5.888	<0.0001	
Pre high/pre low	1.211	0.315	0.737	0.996	
Pre high/during low	1.734	0.447	2.135	0.393	
During high/pre low	1.594	0.411	1.809	0.614	
During high/during low	2.281	0.583	3.230	0.028	
Pre low/during low	1.431	0.072	7.102	<0.0001	

Estimated marginal means and pairwise comparisons are presented across all combinations of experimental period (pre, during) and treatment (condition: low, high; control feeder 1 (c1), control feeder 2 (c2)). Shown are the estimated means, standard errors (SE), 95% confidence intervals (CL) for the estimated means, test statistic and the *P* value for each pairwise comparison.

Table A5
Results of the model examining the effects of the experimental density manipulation on the local average flock size

	Estimate	SE	*z*	*P*
Intercept	1.643	0.628	2.616	0.009
Period during	1.598	0.303	5.276	<0.0001
Condition c2	0.181	0.683	0.266	0.791
Condition high	1.008	0.708	1.425	0.154
Condition low	1.082	0.71	1.523	0.128
Experimental day	−0.006	0.007	−0.807	0.419
Period during:condition c2	−0.917	0.449	−2.044	0.041
Period during:condition high	−0.758	0.343	−2.212	0.027
Period during:condition low	−2.501	0.347	−7.212	<0.0001

Period refers to the experimental period: prior versus during the density manipulation. Condition refers to the different experimental treatments: low = low density, high = high density; c1 and c2 = control feeders. Experimental day describes the day within each experimental period.

Table A6
Estimated marginal means and pairwise comparisons of the local average flock size

Period	Condition	Response	SE	Lower CL	Upper CL
**Estimated marginal means**					
Pre	c1	1.537	0.615	0.329	2.746
During	c1	3.135	0.569	2.018	4.251
Pre	c2	1.718	0.632	0.477	2.960
During	c2	2.399	0.568	1.284	3.514
Pre	High	2.545	0.349	1.860	3.230
During	High	3.385	0.330	2.738	4.032
Pre	Low	2.619	0.355	1.922	3.316
During	Low	1.716	0.334	1.060	2.372
**Pairwise comparisons**					
Comparison	Ratio	SE	*t*	*P*	
Pre c1/during c1	−1.598	0.303	−5.276	<0.0001	
Pre c1/pre c2	−0.181	0.683	−0.266	1.000	
Pre c1/during c2	−0.862	0.624	−1.380	0.866	
Pre c1/pre high	−1.008	0.708	−1.425	0.846	
Pre c1/during high	−1.848	0.698	−2.647	0.142	
Pre c1/pre low	−1.082	0.710	−1.523	0.795	
Pre c1/during low	−0.179	0.701	−0.255	1.000	
During c1/pre c2	1.416	0.642	2.207	0.349	
During c1/during c2	0.736	0.578	1.274	0.908	
During c1/pre high	0.590	0.667	0.884	0.987	
During c1/during high	−0.250	0.657	−0.381	1.000	
During c1/pre low	0.516	0.671	0.770	0.995	
During c1/during low	1.419	0.659	2.153	0.382	
Pre c2/during c2	−0.680	0.334	−2.037	0.458	
Pre c2/pre high	−0.827	0.722	−1.144	0.947	
Pre c2/during high	−1.666	0.713	−2.339	0.274	
Pre c2/pre low	−0.900	0.725	−1.242	0.919	
Pre c2/during low	0.003	0.716	0.004	1.000	
During c2/pre high	−0.146	0.667	−0.219	1.000	
During c2/during high	−0.986	0.656	−1.503	0.806	
During c2/pre low	−0.220	0.670	−0.328	1.000	
During c2/during low	0.683	0.659	1.037	0.969	
Pre high/during high	−0.840	0.163	−5.148	<0.0001	
Pre high/pre low	−0.074	0.379	−0.194	1.000	
Pre high/during low	0.829	0.360	2.303	0.294	
During high/pre low	0.766	0.362	2.115	0.406	
During high/during low	1.669	0.341	4.899	<0.0001	
Pre low/during low	0.903	0.174	5.203	<0.0001	

Estimated marginal means and pairwise comparisons are presented across all combinations of experimental period (pre, during) and treatment (condition: low, high; control feeder 1 (c1), control feeder 2 (c2)). Shown are the estimated means, standard errors (SE), 95% confidence intervals (CL) for the estimated means, test statistic and the *P* value for each pairwise comparison.

Table A7
Results of the model examining the effects of the experimental density manipulation on the network density

	Estimate	SE	*t*	*P*
Intercept	0.643	0.076	8.444	<0.0001
Period during	0.048	0.056	0.85	0.395
Condition c2	0.034	0.075	0.453	0.651
Condition high	−0.013	0.086	−0.145	0.887
Condition low	−0.053	0.087	−0.606	0.554
Experimental day	0	0.001	−0.137	0.891
Period during:condition c2	−0.018	0.082	−0.222	0.825
Period during:condition high	0	0.062	−0.007	0.995
Period during:condition low	−0.197	0.063	−3.121	0.002

Period refers to the experimental period: prior versus during the density manipulation. Condition refers to the different experimental treatments: low = low density, high = high density; c1 and c2 = control feeders. Experimental day describes the day within each experimental period.

Table A8
Estimated marginal means and pairwise comparisons of the network density

Period	Condition	Emmean	SE	Lower CL	Upper CL
**Estimated marginal means**					
Pre	c1	0.641	0.076	0.479	0.802
During	c1	0.688	0.062	0.542	0.834
Pre	c2	0.675	0.081	0.507	0.843
During	c2	0.704	0.062	0.558	0.850
Pre	High	0.628	0.042	0.537	0.719
During	High	0.675	0.036	0.591	0.760
Pre	Low	0.588	0.043	0.496	0.680
During	Low	0.438	0.037	0.353	0.524
**Pairwise comparisons**					
Comparison	Estimate	SE	t.ratio	*P*	
Pre c1/during c1	−0.048	0.056	−0.850	0.990	
Pre c1/pre c2	−0.034	0.075	−0.453	1.000	
Pre c1/during c2	−0.063	0.056	−1.128	0.950	
Pre c1/pre high	0.013	0.086	0.145	1.000	
Pre c1/during high	−0.035	0.085	−0.410	1.000	
Pre c1/pre low	0.053	0.087	0.606	0.998	
Pre c1/during low	0.202	0.085	2.379	0.320	
During c1/pre c2	0.014	0.062	0.223	1.000	
During c1/during c2	−0.016	0.033	−0.481	1.000	
During c1/pre high	0.060	0.075	0.802	0.988	
During c1/during high	0.013	0.071	0.182	1.000	
During c1/pre low	0.100	0.076	1.327	0.867	
During c1/during low	0.250	0.072	3.479	0.102	
Pre c2/during c2	−0.030	0.062	−0.477	1.000	
Pre c2/pre high	0.046	0.090	0.515	0.999	
Pre c2/during high	−0.001	0.089	−0.009	1.000	
Pre c2/pre low	0.087	0.091	0.954	0.976	
Pre c2/during low	0.236	0.089	2.658	0.202	
During c2/pre high	0.076	0.075	1.011	0.961	
During c2/during high	0.029	0.071	0.402	1.000	
During c2/pre low	0.116	0.076	1.534	0.775	
During c2/during low	0.266	0.072	3.696	0.078	
Pre high/during high	−0.047	0.031	−1.529	0.792	
Pre high/pre low	0.040	0.036	1.101	0.956	
Pre high/during low	0.190	0.032	5.992	<0.0001	
During high/pre low	0.087	0.032	2.720	0.119	
During high/during low	0.237	0.021	11.027	<0.0001	
Pre low/during low	0.150	0.033	4.572	<0.0001	

Estimated marginal means and pairwise comparisons are presented across all combinations of experimental period (pre, during) and treatment (condition: low, high; control feeder 1 (c1), control feeder 2 (c2)). Shown are the estimated means, standard errors (SE), 95% confidence intervals (CL) for the estimated means, test statistic and the *P* value for each pairwise comparison.

Table A9
Results of the model examining the effects of the experimental density manipulation on the average edge weight

	Estimate	SE	*t*	*P*
Intercept	6.524	0.655	9.96	<0.0001
Period during	1.072	0.585	1.832	0.067
Condition c2	−0.504	0.782	−0.644	0.52
Condition high	0.101	0.739	0.137	0.892
Condition low	−0.373	0.745	−0.502	0.62
Experimental day	0.015	0.008	1.943	0.052
Period during:condition c2	−1.248	0.854	−1.462	0.144
Period during:condition high	−1.141	0.649	−1.758	0.079
Period during:condition low	−1.787	0.66	−2.708	0.007

Period refers to the experimental period: prior versus during the density manipulation. Condition refers to the different experimental treatments: low = low density, high = high density; c1 and c2 = control feeders. Experimental day describes the day within each experimental period.

Table A10
Estimated marginal means and pairwise comparisons of the average edge weight

Period	Condition	Response	SE	Lower CL	Upper CL
**Estimated marginal means**					
Pre	c1	6.800	0.658	5.461	8.139
During	c1	7.872	0.461	6.813	8.932
Pre	c2	6.296	0.711	4.863	7.730
During	c2	6.120	0.462	5.060	7.181
Pre	High	6.901	0.357	6.167	7.635
During	High	6.833	0.273	6.215	7.450
Pre	Low	6.426	0.369	5.672	7.181
During	Low	5.712	0.285	5.081	6.342
**Pairwise comparisons**					
Comparison	Estimate	SE	*t*	*P*	
Pre c1/during c1	−1.072	0.586	−1.832	0.599	
Pre c1/pre c2	0.504	0.783	0.644	0.998	
Pre c1/during c2	0.680	0.587	1.158	0.943	
Pre c1/pre high	−0.101	0.739	−0.136	1.000	
Pre c1/during high	−0.033	0.716	−0.046	1.000	
Pre c1/pre low	0.373	0.745	0.501	1.000	
Pre c1/during low	1.088	0.720	1.512	0.794	
During c1/pre c2	1.576	0.645	2.445	0.222	
During c1/during c2	1.752	0.342	5.120	<0.0001	
During c1/pre high	0.972	0.587	1.655	0.713	
During c1/during high	1.040	0.534	1.945	0.559	
During c1/pre low	1.446	0.594	2.433	0.304	
During c1/during low	2.161	0.541	3.994	0.043	
Pre c2/during c2	0.176	0.646	0.272	1.000	
Pre c2/pre high	−0.605	0.786	−0.769	0.994	
Pre c2/during high	−0.536	0.765	−0.701	0.996	
Pre c2/pre low	−0.130	0.792	−0.164	1.000	
Pre c2/during low	0.584	0.768	0.761	0.994	
During c2/pre high	−0.781	0.588	−1.326	0.872	
During c2/during high	−0.712	0.535	−1.331	0.865	
During c2/pre low	−0.306	0.596	−0.514	0.999	
During c2/during low	0.408	0.542	0.754	0.992	
Pre high/during high	0.068	0.323	0.211	1.000	
Pre high/pre low	0.474	0.381	1.246	0.918	
Pre high/during low	1.189	0.331	3.597	0.008	
During high/pre low	0.406	0.336	1.210	0.929	
During high/during low	1.121	0.224	4.997	<0.0001	
Pre low/during low	0.715	0.342	2.092	0.421	

Estimated marginal means and pairwise comparisons are presented across all combinations of experimental period (pre, during) and treatment (condition: low, high; control feeder 1 (c1), control feeder 2 (c2)). Shown are the estimated means, standard errors (SE), 95% confidence intervals (CL) for the estimated means, test statistic and the *P* value for each pairwise comparison.

Table A11
Results of the model examining the effects of the experimental density manipulation on the clustering coefficient

	Estimate	SE	*z*	*P*
Intercept	0.632	0.087	7.27	<0.0001
Period during	0.04	0.062	0.638	0.524
Condition c2	0.019	0.083	0.231	0.817
Condition high	−0.061	0.099	−0.614	0.549
Condition low	−0.033	0.099	−0.337	0.741
Experimental day	0	0.001	0.418	0.676
Period during:condition c2	−0.016	0.091	−0.177	0.86
Period during:condition high	0.048	0.069	0.695	0.488
Period during:condition low	−0.228	0.07	−3.256	0.001

Period refers to the experimental period: prior versus during the density manipulation. Condition refers to the different experimental treatments: low = low density, high = high density; c1 and c2 = control feeders. Experimental day describes the day within each experimental period.

Table A12
Estimated marginal means and pairwise comparisons of the clustering coefficient

Period	Condition	Response	SE	Lower CL	Upper CL
**Estimated marginal means**					
Pre	c1	0.638	0.087	0.452	0.824
During	c1	0.677	0.072	0.508	0.847
Pre	c2	0.657	0.092	0.465	0.849
During	c2	0.681	0.072	0.511	0.850
Pre	High	0.577	0.048	0.473	0.682
During	High	0.665	0.042	0.566	0.763
Pre	Low	0.604	0.049	0.498	0.710
During	Low	0.416	0.043	0.317	0.515
**Pairwise comparisons**					
Comparison	Odds ratio	SE	*t*	*P*	
Pre c1/during c1	−0.040	0.062	−0.638	0.998	
Pre c1/pre c2	−0.019	0.083	−0.231	1.000	
Pre c1/during c2	−0.043	0.062	−0.686	0.997	
Pre c1/pre high	0.061	0.099	0.614	0.998	
Pre c1/during high	−0.027	0.097	−0.275	1.000	
Pre c1/pre low	0.033	0.099	0.337	1.000	
Pre c1/during low	0.222	0.097	2.281	0.369	
During c1/pre c2	0.020	0.068	0.299	1.000	
During c1/during c2	−0.003	0.036	−0.087	1.000	
During c1/pre high	0.100	0.087	1.158	0.925	
During c1/during high	0.013	0.083	0.157	1.000	
During c1/pre low	0.073	0.087	0.838	0.985	
During c1/during low	0.261	0.083	3.141	0.154	
Pre c2/during c2	−0.024	0.069	−0.344	1.000	
Pre c2/pre high	0.080	0.103	0.776	0.992	
Pre c2/during high	−0.007	0.101	−0.074	1.000	
Pre c2/pre low	0.053	0.103	0.509	0.999	
Pre c2/during low	0.241	0.101	2.377	0.316	
During c2/pre high	0.103	0.087	1.193	0.914	
During c2/during high	0.016	0.083	0.194	1.000	
During c2/pre low	0.076	0.087	0.873	0.981	
During c2/during low	0.265	0.083	3.177	0.147	
Pre high/during high	−0.087	0.034	−2.554	0.175	
Pre high/pre low	−0.027	0.040	−0.675	0.998	
Pre high/during low	0.161	0.035	4.593	<0.001	
During high/pre low	0.060	0.036	1.690	0.694	
During high/during low	0.248	0.024	10.440	<0.001	
Pre low/during low	0.188	0.036	5.197	<0.001	

Estimated marginal means and pairwise comparisons are presented across all combinations of experimental period (pre, during) and treatment (condition: low, high; control feeder 1 (c1), control feeder 2 (c2)). Shown are the estimated means, standard errors (SE), 95% confidence intervals (CL) for the estimated means, test statistic and the *P* value for each pairwise comparison.

Table A13
Results of the model examining whether individuals significantly increased visits to the feeder to which they had been assigned

	Estimate	SE	*z*	*P*
(Intercept)	1.033	0.273	3.788	<0.001
Period during	2.636	0.016	165.087	<0.001
Condition low	−3.509	0.196	−17.931	<0.001
Period during:condition low	−1.521	0.033	−45.742	<0.001

Period refers to the experimental period: prior versus during the density manipulation. Condition refers to the treatment individuals to which had been assigned: low = low density, high = high density.

Table A15
Results of the model examining the effects of the experimental density manipulation on the average flock size experienced by each individual

	Estimate	SE	*t*	*P*
Intercept	2.701	0.891	3.032	0.022
Period during	1.343	0.208	6.446	<0.0001
Condition high	1.303	1.029	1.267	0.251
Condition low	1.357	1.035	1.312	0.235
Period during:condition high	−0.649	0.238	−2.724	0.007
Period during: condition low	−1.994	0.286	−6.963	<0.0001

Period refers to the experimental period: prior versus during the density manipulation. Condition refers to the treatment to which individuals had been assigned: low = low density, high = high density.

Table A14
Estimated marginal means and pairwise comparisons of the proportion of recordings to the feeder to which individuals had been assigned

Period	Condition	Probability	SE	Lower CL	Upper CL
**Estimated marginal means**					
Pre	High	0.737	0.053	0.622	0.827
During	High	0.975	0.007	0.958	0.985
Pre	Low	0.077	0.021	0.045	0.131
During	Low	0.204	0.048	0.125	0.315
**Pairwise comparisons**					
Comparison	Odds ratio	SE	*z*	*P*	
Pre high/during high	0.072	0.001	−165.087	<0.0001	
Pre high/pre low	33.431	6.543	17.931	<0.0001	
Pre high/during low	10.963	2.140	12.266	<0.0001	
During high/pre low	466.626	91.336	31.397	<0.0001	
During high/during low	153.020	29.875	25.766	<0.0001	
Pre low/during low	0.328	0.010	−38.224	<0.0001	

Estimated marginal means and pairwise comparisons are presented across all combinations of experimental period (pre, during) and treatment (condition: low, high). Shown are the estimated means, standard errors (SE), 95% confidence intervals (CL) for the estimated means, test statistic and the *P* value for each pairwise comparison.

Table A16
Estimated marginal means and pairwise comparisons of the average flock size each individual experienced

Period	Condition	Probability	SE	Lower CL	Upper CL
**Estimated marginal means**					
Pre	Control	2.701	0.891	0.538	4.864
During	Control	4.044	0.891	1.881	6.208
Pre	High	4.004	0.514	2.755	5.253
During	High	4.699	0.514	3.450	5.947
Pre	Low	4.058	0.526	2.806	5.310
During	Low	3.407	0.526	2.155	4.659

Pairwise comparisons

Comparison	Odds ratio	SE	*t*	*P*
Pre control/during control	−1.343	0.208	−6.446	<0.0001
Pre control/pre high	−1.303	1.029	−1.267	0.793
Pre control/during high	−1.998	1.029	−1.942	0.458
Pre control/pre low	−1.357	1.035	−1.312	0.772
Pre control/during low	−0.706	1.035	−0.683	0.978
During control/pre high	0.040	1.029	0.039	1.000
During control/during high	−0.654	1.029	−0.636	0.984
During control/pre low	−0.014	1.035	−0.013	1.000
During control/during low	0.637	1.035	0.616	0.986
Pre high/during high	−0.694	0.116	−6.005	<0.0001
Pre high/pre low	−0.054	0.163	−0.331	0.999
Pre high/during low	0.597	0.163	3.653	0.004
During high/pre low	0.640	0.163	3.917	0.002
During high/during low	1.291	0.163	7.901	<0.0001
Pre low/during low	0.651	0.196	3.313	0.014

Estimated marginal means and pairwise comparisons are presented across all combinations of experimental period (pre, during) and treatment (condition: low, high, control). Shown are the estimated means, standard errors (SE), 95% confidence intervals (CL) for the estimated means, test statistic and the *P* value for each pairwise comparison.

Table A17
Results of the model examining the effects of the experimental density manipulation on individuals' weighted degree

	Estimate	SE	*t*	*P*
Intercept	1.473	0.436	3.376	0.013
Period during	0.800	0.171	4.681	<0.001
Condition high	0.643	0.504	1.276	0.245
Condition low	0.799	0.513	1.559	0.163
Period during:condition high	−0.403	0.195	−2.064	0.040
Period during: condition low	−1.491	0.235	−6.346	<0.001

Period refers to the experimental period: prior versus during the density manipulation. Condition refers to the treatment to which individuals had been assigned: low = low density, high = high density.

Table A18
Estimated marginal means and pairwise comparisons of individuals' weighted degree

Period	Condition	Emmean	SE	Lower CL	Upper CL
**Estimated marginal means**					
Pre	Control	1.473	0.436	0.429	2.517
During	Control	2.273	0.436	1.229	3.317
Pre	High	2.115	0.252	1.512	2.718
During	High	2.512	0.252	1.909	3.115
Pre	Low	2.272	0.269	1.659	2.885
During	Low	1.581	0.269	0.968	2.194
**Pairwise comparisons**					
Comparison	Estimate	SE	*t*	*P*	
Pre control/during control	−0.800	0.171	−4.681	<0.0001	
Pre control/pre high	−0.643	0.504	−1.275	0.789	
Pre control/during high	−1.039	0.504	−2.063	0.401	
Pre control/pre low	−0.799	0.513	−1.559	0.644	
Pre control/during low	−0.109	0.513	−0.212	1.000	
During control/pre high	0.158	0.504	0.313	0.999	
During control/during high	−0.239	0.504	−0.475	0.996	
During control/pre low	0.001	0.513	0.002	1.000	
During control/during low	0.692	0.513	1.349	0.754	
Pre high/during high	−0.397	0.095	−4.184	0.001	
Pre high/pre low	−0.157	0.142	−1.106	0.879	
Pre high/during low	0.534	0.142	3.773	0.003	
During high/pre low	0.240	0.142	1.697	0.535	
During high/during low	0.931	0.142	6.575	<0.0001	
Pre low/during low	0.691	0.161	4.285	<0.0001	

Estimated marginal means and pairwise comparisons are presented across all combinations of experimental period (pre, during) and treatment (condition: low, high, control). Shown are the estimated means, standard errors (SE), 95% confidence intervals (CL) for the estimated means, test statistic and the *P* value for each pairwise comparison. *P* values are inferred from 10 000 random permutations.

Table A19
Results of the model examining the effects of the experimental density manipulation on individuals' average edge weight

	Estimate	SE	*t*	*P*
Intercept	0.076	0.009	8.489	<0.001
Period during	−0.020	0.003	−5.903	<0.001
Condition high	−0.009	0.010	−0.908	0.396
Condition low	−0.006	0.011	−0.576	0.582
Period during:condition high	0.015	0.004	4.049	<0.001
Period during: condition low	−0.007	0.005	−1.440	0.152

Period refers to the experimental period: prior versus during the density manipulation. Condition refers to the treatment to which individuals had been assigned: low = low density, high = high density.

Table A20
Estimated marginal means and pairwise comparisons of individuals' average edge weight

Period	Condition	Emmean	SE	Lower CL	Upper CL
**Estimated marginal means**					
Pre	Control	0.076	0.009	0.055	0.098
During	Control	0.057	0.009	0.035	0.078
Pre	High	0.067	0.005	0.054	0.079
During	High	0.063	0.005	0.050	0.075
Pre	Low	0.070	0.006	0.057	0.083
During	Low	0.044	0.006	0.031	0.057
**Pairwise comparisons**					
Comparison	Estimate	SE	*t*	*P*	
Pre control/during control	0.020	0.003	5.903	0.846	
Pre control/pre high	0.009	0.010	0.908	0.345	
Pre control/during high	0.014	0.010	1.314	1.000	
Pre control/pre low	0.006	0.011	0.576	0.812	
Pre control/during low	0.032	0.011	3.052	0.123	
During control/pre high	−0.010	0.010	−0.978	0.655	
During control/during high	−0.006	0.010	−0.572	0.990	
During control/pre low	−0.013	0.011	−1.278	0.789	
During control/during low	0.013	0.011	1.198	0.826	
Pre high/during high	0.004	0.002	2.293	0.202	
Pre high/pre low	−0.003	0.003	−1.166	0.273	
Pre high/during low	0.023	0.003	7.962	<0.001	
During high/pre low	−0.008	0.003	−2.639	0.908	
During high/during low	0.019	0.003	6.489	<0.001	
Pre low/during low	0.026	0.003	8.360	<0.001	

Estimated marginal means and pairwise comparisons are presented across all combinations of experimental period (pre, during) and treatment (condition: low, high, control). Shown are the estimated means, standard errors (SE), 95% confidence intervals (CL) for the estimated means, test statistic and the *P* value for each pairwise comparison. *P* values are inferred from 10 000 random permutations.

Table A21
Results of the model examining the effects of the experimental density manipulation on individuals' weighted clustering coefficient

	Estimate	SE	*z*	*P*
Intercept	2.486	0.367	6.783	<0.001
Period during	−0.052	0.126	−0.416	0.677
Condition high	0.127	0.424	0.301	0.764
Condition low	0.214	0.430	0.497	0.620
Period during:condition high	−0.505	0.144	−3.499	<0.001
Period during: condition low	−0.446	0.169	−2.638	0.008

Period refers to the experimental period: prior versus during the density manipulation. Condition refers to the treatment to which individuals had been assigned: low = low density, high = high density.

Table A22
Estimated marginal means and pairwise comparisons of individuals' weighted clustering coefficient

Period	Condition	Response	SE	Lower CL	Upper CL
**Estimated marginal means**					
Pre	Control	0.923	0.026	0.854	0.961
During	Control	0.919	0.027	0.848	0.959
Pre	High	0.932	0.014	0.900	0.954
During	High	0.887	0.021	0.838	0.922
Pre	Low	0.937	0.013	0.905	0.958
During	Low	0.900	0.020	0.855	0.933
**Pairwise comparisons**					
Comparison	Odds ratio	SE	*t*	*P*	
Pre control/during control	1.054	0.132	0.416	0.972	
Pre control/pre high	0.880	0.373	−0.301	1.000	
Pre control/during high	1.538	0.650	1.019	0.912	
Pre control/pre low	0.808	0.347	−0.497	0.996	
Pre control/during low	1.329	0.568	0.665	0.986	
During control/pre high	0.836	0.353	−0.425	0.998	
During control/during high	1.459	0.615	0.897	0.947	
During control/pre low	0.766	0.329	−0.620	0.990	
During control/during low	1.261	0.538	0.544	0.994	
Pre high/during high	1.747	0.124	7.848	<0.001	
Pre high/pre low	0.917	0.102	−0.776	0.971	
Pre high/during low	1.509	0.154	4.041	0.001	
During high/pre low	0.525	0.056	−6.036	<0.001	
During high/during low	0.864	0.083	−1.522	0.650	
Pre low/during low	1.645	0.187	4.388	<0.001	

Estimated marginal means and pairwise comparisons are presented across all combinations of experimental period (pre, during) and treatment (condition: low, high, control). Shown are the estimated means, standard errors (SE), 95% confidence intervals (CL) for the estimated means, test statistic and the *P* value for each pairwise comparison. *P* values are inferred from 10 000 random permutations.

Table A23
Results of the model examining the effects of the experimental density manipulation on individuals' weighted eigenvector centrality

	Estimate	SE	*z*	*P*
Intercept	0.633	0.058	10.873	<0.0001
Period during	−0.004	0.058	−0.073	0.942
Condition high	−0.063	0.067	−0.943	0.346
Condition low	−0.012	0.075	−0.160	0.873
Period during:condition high	0.036	0.066	0.551	0.581
Period during: condition low	−0.346	0.079	−4.374	<0.0001

Period refers to the experimental period: prior versus during the density manipulation. Condition refers to the treatment to which individuals had been assigned: low = low density, high = high density.

Table A24
Estimated marginal means and pairwise comparisons of individuals' weighted eigenvector centrality

Period	Condition	Response	SE	Lower CL	Upper CL
**Estimated marginal means**					
Pre	Control	0.633	0.058	0.519	0.748
During	Control	0.629	0.058	0.514	0.743
Pre	High	0.570	0.033	0.504	0.636
During	High	0.602	0.033	0.536	0.668
Pre	Low	0.621	0.048	0.527	0.715
During	Low	0.270	0.048	0.176	0.365

Pairwise comparisons

Comparison	Odds ratio	SE	*t*	*P*
Pre control/during control	0.004	0.058	0.073	1.000
Pre control/pre high	0.063	0.067	0.943	0.935
Pre control/during high	0.031	0.067	0.464	0.997
Pre control/pre low	0.012	0.075	0.160	1.000
Pre control/during low	0.363	0.075	4.807	<0.0001
During control/pre high	0.059	0.067	0.880	0.951
During control/during high	0.027	0.067	0.401	0.999
During control/pre low	0.008	0.075	0.104	1.000
During control/during low	0.359	0.075	4.751	<0.0001
Pre high/during high	−0.032	0.032	−1.005	0.916
Pre high/pre low	−0.051	0.050	−1.022	0.910
Pre high/during low	0.299	0.050	5.976	<0.0001
During high/pre low	−0.019	0.050	−0.381	0.999
During high/during low	0.332	0.050	6.617	<0.0001
Pre low/during low	0.351	0.054	6.453	<0.0001

Estimated marginal means and pairwise comparisons are presented across all combinations of experimental period (pre, during) and treatment (condition: low, high, control). Shown are the estimated means, standard errors (SE), 95% confidence intervals (CL) for the estimated means, test statistic and the *P* value for each pairwise comparison. *P* values are inferred from 10 000 random permutations.

Table A25
Results of the model examining the effects of the experimental density manipulation on each individuals' probability to discover a novel food patch

	Estimate	SE	*z*	*P*
Intercept	1.603	0.644	2.488	0.013
Period during	−1.284	0.616	−2.086	0.037
Condition high	−1.119	0.736	−1.519	0.129
Condition low	−0.421	0.825	−0.511	0.610
Period during:condition high	3.698	1.002	3.690	<0.0001
Period during: condition low	1.028	0.986	1.042	0.297

Period refers to the experimental period: prior versus during the density manipulation. Condition refers to the treatment to which individuals had been assigned: low = low density, high = high density.

Table A26
Estimated marginal means and pairwise comparisons of individuals' likelihood of discovering a novel food source

Period	Condition	Probability	SE	Lower CL	Upper CL
**Estimated marginal means**					
During	Control	0.579	0.144	0.301	0.814
Pre	Control	0.832	0.090	0.583	0.946
During	High	0.948	0.039	0.793	0.988
Pre	High	0.619	0.085	0.445	0.767
During	Low	0.716	0.134	0.408	0.902
Pre	low	0.765	0.094	0.538	0.901
**Pairwise comparisons**					
Comparison	Odds ratio	SE	*t*	*P*	
Pre control/during control	0.277	0.170	−2.086	0.299	
Pre control/pre high	0.076	0.075	−2.621	0.097	
Pre control/during high	0.847	0.584	−0.240	1.000	
Pre control/pre low	0.545	0.483	−0.685	0.983	
Pre control/during low	0.422	0.331	−1.100	0.881	
During control/pre high	0.274	0.279	−1.272	0.800	
During control/during high	3.061	2.254	1.519	0.652	
During control/pre low	1.970	1.822	0.733	0.978	
During control/during low	1.524	1.257	0.511	0.996	
Pre high/during high	11.171	8.834	3.052	0.030	
Pre high/pre low	7.189	6.856	2.068	0.308	
Pre high/during low	5.562	4.916	1.941	0.380	
During high/pre low	0.643	0.431	−0.658	0.986	
During high/during low	0.498	0.259	−1.338	0.763	
Pre low/during low	0.774	0.599	−0.331	0.999	

Estimated marginal means and pairwise comparisons are presented across all combinations of experimental period (pre, during) and treatment (condition: low, high, control). Shown are the estimated means, standard errors (SE), 95% confidence intervals (CL) for the estimated means, test statistic and the *P* value for each pairwise comparison.

Table A27
Results of the model examining the effects of the experimental density manipulation on each individual's order of discovering a novel food patch

	Estimate	SE	*z*	*P*
Intercept	1.317	0.240	5.484	<0.0001
Period during	−0.269	0.234	−1.150	0.250
Condition high	0.105	0.289	0.362	0.717
Condition low	−0.178	0.310	−0.575	0.565
Period during:condition high	0.646	0.283	2.282	0.022
Period during: condition low	0.953	0.349	2.726	0.006

Period refers to the experimental period: prior versus during the density manipulation. Condition refers to the treatment to which individuals had been assigned: low = low density, high = high density.

Table A28
Estimated marginal means and pairwise comparisons of individuals' order of discovering a novel food source

Period	Condition	Response	SE	Lower CL	Upper CL
**Estimated marginal means**					
Pre	Control	3.732	0.896	2.322	5.996
During	Control	2.851	0.763	1.680	4.838
Pre	High	4.143	0.666	3.016	5.692
During	High	6.042	1.019	4.330	8.430
Pre	Low	3.122	0.614	2.117	4.605
During	Low	6.184	1.453	3.888	9.836
**Pairwise comparisons**					
Comparison	Ratio	SE	*t*	*P*	
Pre control/during control	1.309	0.306	1.150	0.859	
Pre control/pre high	0.901	0.260	−0.362	0.999	
Pre control/during high	0.618	0.181	−1.642	0.572	
Pre control/pre low	1.195	0.370	0.575	0.993	
Pre control/during low	0.603	0.203	−1.503	0.663	
During control/pre high	0.688	0.215	−1.197	0.838	
During control/during high	0.472	0.149	−2.373	0.172	
During control/pre low	0.913	0.303	−0.274	1.000	
During control/during low	0.461	0.164	−2.174	0.256	
Pre high/during high	0.686	0.110	−2.358	0.178	
Pre high/pre low	1.327	0.260	1.444	0.700	
Pre high/during low	0.670	0.160	−1.681	0.547	
During high/pre low	1.935	0.405	3.158	0.023	
During high/during low	0.977	0.230	−0.099	1.000	
Pre low/during low	0.505	0.131	−2.631	0.096	

Estimated marginal means and pairwise comparisons are presented across all combinations of experimental period (pre, during) and treatment (condition: low, high, control). Shown are the estimated means, standard errors (SE), 95% confidence intervals (CL) for the estimated means, test statistic and the *P* value for each pairwise comparison.

Table A29
Results of the model examining the effects of the experimental density manipulation on each individual's latency to discover a novel food patch

	Estimate	SE	*z*	*P*
Intercept	18.799	1.127	16.685	<0.001
Period during	−1.272	1.006	−1.265	0.206
Condition high	−2.378	1.342	−1.772	0.076
Condition low	−4.145	1.423	−2.913	0.004
Period pre:condition high	1.211	1.218	0.994	0.320
Period pre:condition low	3.965	1.493	2.657	0.008

Period refers to the experimental period: prior versus during the density manipulation. Condition refers to the treatment to which individuals had been assigned: low = low density, high = high density.

Table A30
Estimated marginal means and pairwise comparisons of individuals' latency of discovering a novel food source

Period	Condition	Response	SE	Lower CL	Upper CL
**Estimated marginal means**					
Pre	Control	353.387	42.359	274.665	442.016
During	Control	307.17	43.216	227.734	398.469
Pre	High	269.626	24.1	224.121	319.334
During	High	267.61	25.246	220.063	319.805
Pre	Low	214.735	25.593	167.155	268.266
During	Low	300.919	35.815	234.328	375.827
**Pairwise comparisons**					
Comparison	Ratio	SE	*t*	*P*	
Pre control/during control	1.272	1.006	1.265	0.803	
Pre control/pre high	2.378	1.342	1.772	0.487	
Pre control/during high	2.440	1.366	1.785	0.478	
Pre control/pre low	4.145	1.423	2.913	0.046	
Pre control/during low	1.452	1.528	0.950	0.933	
During control/pre high	1.106	1.434	0.771	0.972	
During control/during high	1.167	1.456	0.802	0.967	
During control/pre low	2.872	1.508	1.904	0.404	
During control/during low	0.179	1.608	0.112	1.000	
Pre high/during high	0.061	0.688	0.089	1.000	
Pre high/pre low	1.766	0.820	2.154	0.266	
Pre high/during low	−0.927	1.002	−0.924	0.940	
During high/pre low	1.705	0.886	1.924	0.392	
During high/during low	−0.988	0.993	−0.996	0.919	
Pre low/during low	−2.693	1.105	−2.438	0.150	

Estimated marginal means and pairwise comparisons are presented across all combinations of experimental period (pre, during) and treatment (condition: low, high, control). Shown are the estimated means, standard errors (SE), 95% confidence intervals (CL) for the estimated means, test statistic and the *P* value for each pairwise comparison.

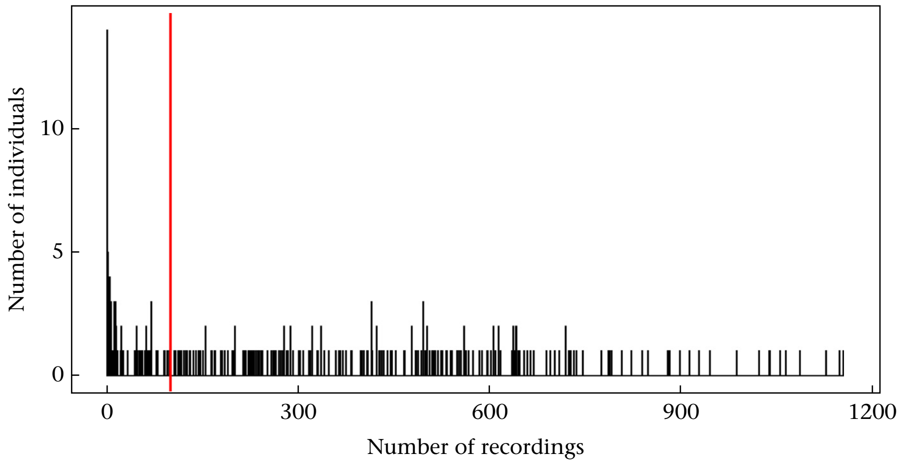

Figure A1. Distribution of the number of individuals by the number of recordings during the pre-experimental period. The vertical red line shows the cut-off at 100 recordings for individuals to be included in the analysis. From all 259 individuals that visited a feeder, 75% had been recorded at least 100 times (minimum = 106, mean = 467, maximum = 1154, SD = 247.88). The excluded 25% of birds had been recorded on average only 27 times (minimum = 1, maximum = 98, SD = 29.83) during the pre-experimental period.

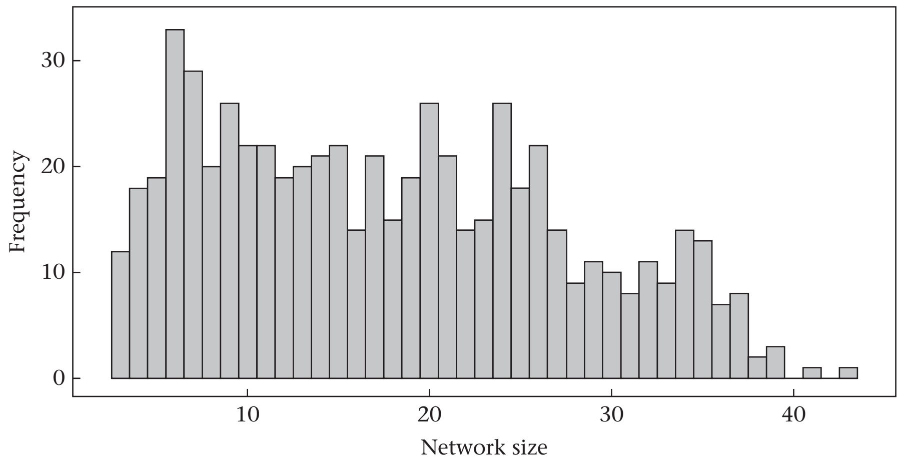

Figure A2. Distribution of network sizes inferred for the feeder locations.

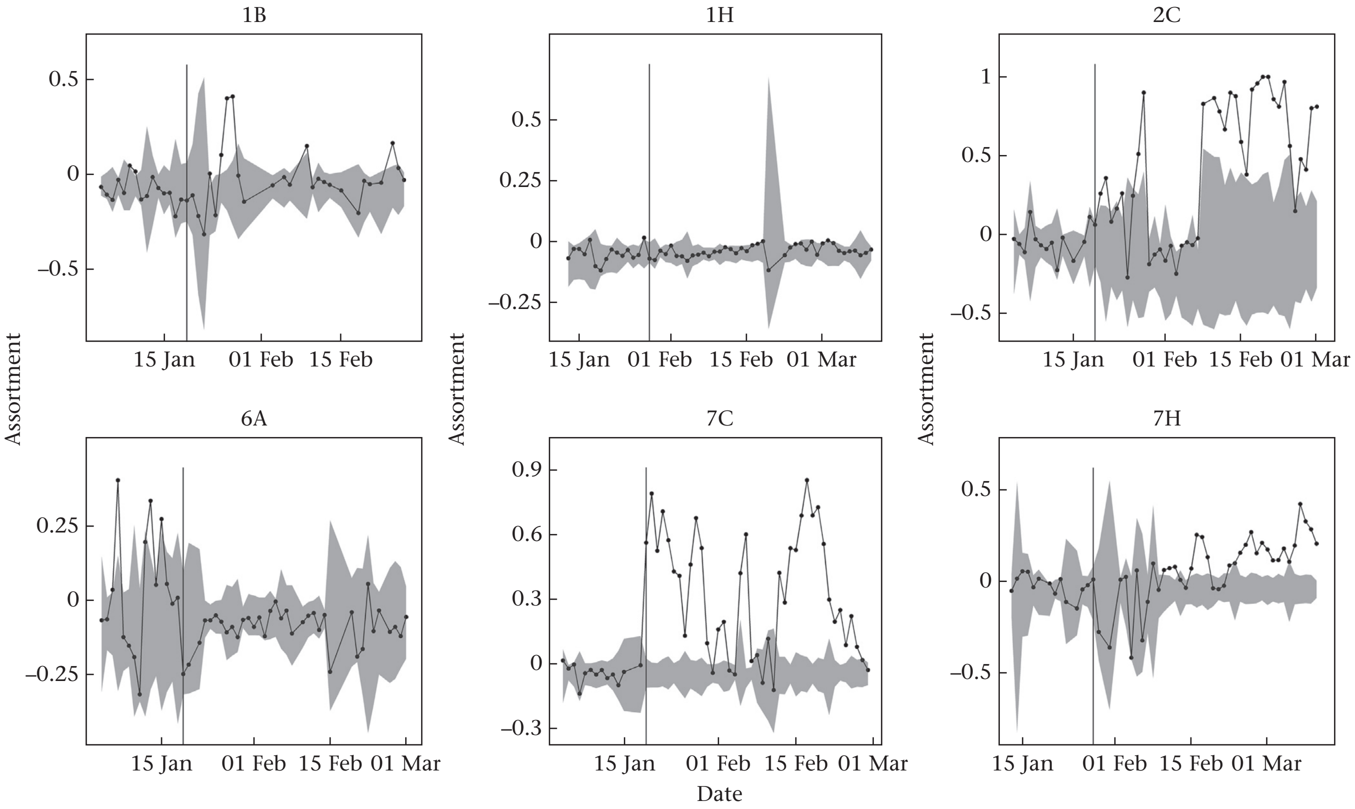

Figure A3. Daily assortativity coefficients for each network shown as black dots and lines. Grey shading represents 95% range of coefficients generated from 1000 random networks. Vertical black line shows the start of the density manipulation.
